# Does Intrarectal Administration of *Christensenella minuta* DSM22607 Impact Body Weight?

**DOI:** 10.3390/nu18101593

**Published:** 2026-05-17

**Authors:** Dorottya Zsálig, Ádám Molnár, Monika Kerényi, Fruzsina Péter, Gellért Gerencsér, Éva Polyák

**Affiliations:** 1 Doctoral School of Health Sciences, Faculty of Health Sciences, University of Pécs, 7621 Pécs, Hungary; 2 Preclinical Research Center, Medical School, University of Pécs, 7624 Pécs, Hungary; molnar.adam2@aok.pte.hu (Á.M.); gerencser.gellert@aok.pte.hu (G.G.); 3 Department of Medical Microbiology and Immunology, Faculty of Medicine, University of Pécs, Szigeti út 12, 7624 Pécs, Hungary; kerenyi.monika@pte.hu; 4 Institute of Nutritional Science and Dietetics, Faculty of Health Sciences, University of Pécs, Vörösmarty út 3, 7621 Pécs, Hungary; fruzsina.peter@etk.pte.hu; 5 Department of Public Health Medicine, Medical School, University of Pécs, 7624 Pécs, Hungary

**Keywords:** obesity, gut microbiome, *Christensenella minuta*, weight regulation, probiotics

## Abstract

Background: *Christensenella minuta* (*C. minuta*) is a promising next-generation probiotic linked to reduced body weight, inhibition of obesogenic processes, and enhanced metabolic profiles. However, the extent and persistence of these effects, particularly under varying dietary conditions, remain uncertain. Objective: This study aimed to examine the effects of intrarectal administration of *C. minuta* on body weight regulation in vivo under different dietary patterns, with or without antibiotic pretreatment, both during the intervention and over the long term. Particular emphasis was placed on exploring the interactions between *C. minuta* supplementation, dietary background, caloric intake, and body weight gain. Methods: A total of 180 CD1 mice (both sexes equally) were allocated into nine experimental groups based on diet, with and without *C. minuta* supplementation, and with and without antibiotic pretreatment. The bacterial suspension was administered intrarectally once a week for three consecutive weeks in the treatment groups. Body weight was monitored weekly, and food intake was recorded biweekly over the 12-week study period. Visceral fat mass was measured postmortem. Results: Groups treated with *C. minuta* with antibiotic pretreatment exhibited significantly lower body weight gain than the control groups during the intervention phase in both sexes, irrespective of caloric intake and dietary pattern, indicating that the reduced weight gain was attributable to the effect of *C. minuta*. Regarding long-term effects following the cessation of administration, sexual dimorphism was observed: while no lasting impact was found in males, the body weight gain inhibiting effect of *C. minuta* treatment persisted in females. Furthermore, females treated with *C. minuta* exhibited the lowest levels of visceral fat among all groups. Caloric intake was not significantly associated with body weight gain at any time point in this study. Conclusions: *C. minuta* exerts a transient, caloric intake-independent inhibitory effect on body weight gain. The absence of sustained effects highlights the necessity for continuous or optimized administration protocols to ensure the attainment of long-term benefits in the future. The results of this study support the hypothesis that *C. minuta* can act as a modulator of host metabolism and body composition, underscoring the significance of treatment duration in this process.

## 1. Introduction

The gut microbiome is a fundamental component of host health and exerts a significant influence on the regulation of the nervous system through the gut–brain axis. It has been proven to have beneficial effects on immune function [1,2], support metabolic health and toxin elimination, and modulate endocrine functions [2]. Furthermore, the composition of the gut microbiota is a contributing factor to the maintenance of a healthy body weight, in addition to protecting against obesogenic factors that lead to obesity [3,4].

Obesity represents an escalating global and individual health concern with a well-documented association with various diseases, including cardiovascular disease, diabetes, and certain cancers [5]. This condition may be mitigated by strategically manipulating the gut microbiota to achieve and sustain normal body weight [4,5].

Certain bacterial strains, such as *Prevotella* spp., *Akkermansia muciniphila*, and *Christensenella minuta*, have been significantly associated with leanness and weight reduction [6,7]. A high diversity of microbiota, particularly including these taxa, has been found to be correlated with lower body weight. It has been demonstrated that an appropriate diet and the intake of probiotics can enhance the prevalence of these bacterial strains [4,8].

The presence of *Christensenellaceae* at higher levels has been consistently observed in individuals with normal body mass across various populations, including Chinese, Japanese, and US cohorts, as well as UK twins and other European groups [9,10,11,12]. *Christensenellaceae*, particularly *C. minuta*, is strongly linked to a lower body mass index (BMI) [13].

Goodrich et al. demonstrated the potential of *Christensenellaceae* to modulate body weight; furthermore, fecal microbiota transplantation enriched with this family resulted in a reduction in weight gain in obese mouse models [12]. Mazier et al. demonstrated that oral supplementation with *C. minuta* DSM33407 in mice effectively mitigated diet-induced obesity. This was achieved by enhancing the metabolic rate, reducing adipose tissue and leptin levels, decreasing resistin levels, and significantly inhibiting hepatic fat synthesis, all without altering the animals’ dietary intake [14]. In murine studies, the administration of *C. minuta* has been shown to significantly mitigate weight gain, particularly in subjects exposed to a high-fat diet [15,16]. Liu et al. demonstrated that the gut bacterium *C. minuta* enhanced host metabolism by producing acylated secondary bile acids. These compounds inhibit farnesoid X receptor (FXR) signaling, resulting in decreased weight gain and fat accumulation in mice subjected to a high-fat diet, thereby suggesting a potential anti-obesity effect. Notably, the levels of these bile acids were found to be lower in individuals with type 2 diabetes, indicating their clinical significance [17]. The administration of live *C. minuta* resulted in increased physical activity, decreased feed efficiency, and enhanced energy loss in the feces of mice. This intervention also elevated the resting metabolic rate, particularly in females. Furthermore, an increase in gut microbial biomass was observed, along with a decrease in microbiota diversity. Overall, *C. minuta* exerted a significant impact on host energy metabolism, even at low relative abundance [18].

Antibiotic pretreatment is frequently utilized in fecal microbiota transplantation to temporarily diminish the complexity of the gut microbiota, thereby establishing a controlled environment for assessing the predominant effects of specific bacterial strains, such as *C. minuta* [17,19,20].

A study examining the interactions of *C. minuta* with other gut microorganisms revealed that it is a functionally active and ecologically significant bacterial species. It has the capacity to modulate the microbiota composition through mechanisms such as cross-feeding, nutrient competition, and metabolic complementation [21]. The mechanisms proposed indicate their potential for targeted therapeutic applications, particularly in the context of metabolic, inflammatory, and obesity-related disorders [22,23].

One of the main objectives of our study was to investigate the proof-of-concept metabolic effects of *C. minuta* under controlled experimental conditions and to ensure reliable delivery of viable bacteria to the colon. The present study aimed to build on the results described above to elucidate the effect of weekly intrarectal administration of *C. minuta* for a period of three weeks on body weight gain in normal and high-fat diets, as well as the consumption of sweetened water, with and without prior antibiotic treatment. Additionally, the present study sought to examine the hypothesis of whether the sweetener exerts any impact on body weight in conjunction with *C. minuta* or through interaction. Furthermore, this study aimed to investigate the long-term sustainability of these effects, thereby evaluating the potential of *C. minuta* as a live biotherapeutic product for the management of obesity and metabolic disorders.

## 2. Materials and Methods

### 2.1. Bacterial Cultivation

The *Christensenella minuta* DSM 22607 strain (Leibniz Institute DSMZ—German Collection of Microorganisms and Cell Cultures GmbH, Braunschweig, Germany) was cultured under strictly anaerobic conditions using modified Gifu Anaerobic Medium (GAM; HiMedia Laboratories GmbH, Einhausen, Germany) in an anaerostat. The cultivation process was conducted at 37.5 °C for four days in an atmosphere of 5% H_2_, 5% CO_2_, and 90% N_2_. In addition, bacterial growth and concentration were carefully monitored prior to administration. Specifically, growth was assessed by measuring optical density at 600 nm (OD600), which is a widely accepted method for estimating bacterial cell density and ensuring consistency across preparations. Species specific identification of the cultured strain was confirmed using Matrix-Assisted Laser Desorption/Ionization Time-of-Flight (MALDI-TOF) mass spectrometry analysis on the Biotyper 3. (Bruker Daltonics, Bremen, Germany) platform. All cultivation procedures were performed at the Department of Medical Microbiology, Medical School, University of Pécs.

### 2.2. Animal Assay

The animal study protocol was approved by the Institutional Review Board of the Food Chain Safety and Animal Health Department, Food Chain Safety and Animal Health Division, Baranya County Government Office (BA02/2000-21/2024; 30 April 2024), Figure S1. All procedures were conducted in accordance with the 3Rs principles (Replacement, Reduction, and Refinement) to minimize animal suffering. The animal experiments were carried out in compliance with the ARRIVE [24] guidelines.

The study employed 180 CD1 mice with an equal distribution of sexes (90 males and 90 females, aged six weeks). The animals were randomly allocated to nine experimental groups, with 20 mice per group. These groups were stratified based on dietary regimen, *C. minuta* administration, prior antibiotic treatment, and gender. Mice on a standard normal diet were divided into three groups according to supplementation: normal diet (ND), normal diet supplemented with *C. minuta* (ND + CM), and normal diet supplemented with *C. minuta* and preliminary antibiotic treatment (ND + AB + CM) Similarly, mice on the high-fat diet were separated into the following groups: high-fat diet (HFD), high-fat diet supplemented with *C. minuta* (HFD + CM), and high-fat diet supplemented with both *C. minuta* and preliminary antibiotic treatment (HFD + AB + CM). In a parallel experiment, the mice were fed a standard diet and provided with sweetened drinking water (SD). These mice were divided into the following groups according to supplementation: sweetener diet (SD), sweetener diet supplemented with *C. minuta* (SD + CM), and sweetener diet supplemented with both *C. minuta* and preliminary antibiotic treatment (SD + AB + CM). All animals were housed in sex-segregated standard cages, with five individuals per cage, and maintained under conventional conditions, temperature (23 ± 2 °C), and controlled luminosity (12 h light/12 h dark) in the same building. Food Chain Safety and Animal Health Department, Food Chain Safety and Animal Health Division, Baranya County Government Office approved this study protocol. Three deaths occurred: two in the HFD male group and one in the SD + CM + AB female group. No specific cause was identified. The detailed group allocations and dietary compositions are summarized in Table 1.

Mice in the ND, ND + CM, ND + CM + AB, SD, SD + CM, and SD + AB + CM groups were maintained on a standard normal diet comprising 9% energy from fat, 24% energy from protein, and 67% energy from carbohydrates (Ssniff, Spezialdiäten GmbH, Soest, Germany Rat/mouse maintenance; 3.225 kcal/kg, 5 g fiber/100 g). In contrast, the HFD, HFD + CM, and HFD + CM + AB groups were provided with a high-fat diet consisting of 18% energy from fat, 24% energy from protein, and 58% energy from carbohydrates (Teklad Global Rodent Diet, Inotiv, Madison, WI, USA (Sterilizable) 2018S; 3.100 kcal/kg, 3.8 g fiber/100 g). Food and water were provided ad libitum twice a week in quantities of 300 mL or g for each group.

Mice in the SD, SD + CM, and SD + AB + CM groups, similar to the other experimental groups, received fresh drinking water twice weekly, in a volume of 300 mL. In each instance, five commercially available sweetener tablets (containing 173.5 mg sodium cyclamate and 46.5 mg sodium saccharin) were dissolved in water. The methodology applied corresponded to a 0.005% concentration, which is significantly lower than the commonly used 0.1–0.3% range reported in the literature [25,26,27,28]. This approach aimed to model a continuous, low, and safe level of intake that reflects the realistic conditions of sugar substitution [29]. All solutions were freshly prepared at the same concentrations.

During the acclimatization period, mice in the antibiotic-treated groups received drinking water supplemented with levofloxacin (a fluoroquinolone) at a concentration of 0.1 mg/mL. This dosage was administered for a duration of 5 days, a period previously shown to be clinically and microbiologically sufficient to significantly deplete the gut microbiota [30,31]. To ensure that the antibiotic did not interfere with the viability of the subsequently administered *C. minuta*, the treatment was followed by a 2-day washout period during which the animals received regular tap water. We closely monitored the animals throughout the pretreatment phase and observed no clinical signs of gastrointestinal distress, such as diarrhea.

The groups receiving *Christensenella minuta* were fasted for 12 h before weekly intrarectal administration. A 0.2 mL suspension of *C. minuta* at a concentration of 10^9^ CFU/mL [14,15] was injected intrarectally once a week for three consecutive weeks. The bacterial suspension was administered intrarectally via a central venous catheter (Certofix^®^ Mono, B. Braun Melsungen AG, Melsungen, Germany) which was inserted to a standardized depth of 2.5 cm into the distal colon lumen to ensure consistent delivery of the bacterial suspension at room temperature, starting on the first day of the experiment. We chose the intrarectal route to deliver *Christensenella minuta* directly to the colon, which is considered its primary ecological niche. This approach allowed us to bypass exposure to gastric acid, bile salts, and digestive enzymes, which could significantly reduce bacterial viability during oral administration.

Subsequent to the administration of bacteria, the mice were maintained on the same diets for a period of eight weeks. The dietary intervention was initiated during the adaptation week in order to monitor the long-term effects of the treatment. Thus, the experiment spanned a total of 12 weeks, including an adaptation period with and without antibiotic pretreatment (1 week), *C. minuta* administration (3 weeks: during weeks 2–4), and a period of long-term observation (8 weeks: weeks 5–12).

Body weight was measured weekly using an analytical scale (Boeco BAS 31 Plus, Boeckel + Co. GmbH + Co. KG, Hamburg, Germany; accuracy ± 0.01 g).

The quantity of food consumed was determined by calculating the difference between the initial 300 g of feed provided to each cage per week and the remaining feed in the cage at the end of each week. The same methodology was used to measure water intake.

The average caloric intake (kcal/g) was calculated according to the manufacturer’s specifications (3.225 kcal/kg for the standard diet and 3.100 kcal/kg for the high-fat diet). Each group was autopsied after 12 weeks, and the visceral adipose tissues were removed and measured using the aforementioned analytical scale.

At the end of the 12th week, all surviving animals (*n* = 177) were euthanized by cervical dislocation. Immediately following euthanasia, visceral adipose tissues were surgically removed, accurately weighed (±0.01 g), and immediately snap frozen and stored at −40 °C for further biochemical assessments (Figure S2).

### 2.3. Statistical Analysis

Sample size was determined to provide sufficient power for a sex-stratified 3 × 3 factorial design. Based on an a priori power analysis for an F-test (ANOVA: Fixed effects, special, main effects and interactions) for each sex, a total sample size of *n* = 90 (*n* = 10 per group) provided a statistical power of 0.93 to detect a large effect size (f = 0.40) for main effects and a power of 0.85 for interaction effects, assuming an alpha level of 0.05. These calculations confirm that the sample size is sufficient to provide robust statistical conclusions while adhering to animal reduction principles.

This balanced design ensures that the interaction between diet and treatment can be robustly analyzed for both males and females separately.

To account for the biological and metabolic differences between sexes, all statistical analyses were performed separately for males and females, resulting in an effective sample size of *n* = 10 per group per sex. This sample size was chosen to provide sufficient statistical power to detect main effects and interactions while adhering to the 3Rs principles of animal reduction.

The normality of the data distribution for each variable was assessed using the Shapiro–Wilk test. Differences among groups in terms of body weight gain, food and energy intake, and fluid consumption were evaluated using either a one-way ANOVA or the Kruskal–Wallis test, contingent upon the distribution. To account for baseline body mass differences among groups, relative body weight gain (%) was employed as the primary outcome measure, as it more effectively controls for potential bias arising from initial weight differences. Correlations between body weight and energy intake were determined through Spearman’s rank correlation analysis, as this non-parametric method is appropriate for data that may not follow a normal distribution.

A 3 × 3 factorial design was utilized to examine the effects and interactions of two independent variables: diet type (standard diet, high-fat diet, and standard diet with sweetener-supplemented drinking water) and treatment (untreated control groups, *Christensenella minuta* treated groups [CM], and antibiotic pretreated and *C. minuta* treated groups [AB + CM]) [32]. Analyses were conducted separately for the bacterial administration phase and the follow-up phase post-discontinuation to capture both immediate and long-term effects. Body weight, energy intake, and fluid consumption were analyzed as dependent variables. A two-way ANOVA was employed for statistical evaluation, conducted separately for females and males due to significant sex-related biological differences in baseline body weight, metabolic profile, and bacterial response. To ensure the robustness of our findings, we prioritized Analysis of Covariance (ANCOVA) to evaluate the effects of treatment while accounting for dietary and sex-related variables. These analyses confirmed that the observed reduction in weight gain was specifically attributable to the bacterial treatment rather than variations in energy consumption or other confounding factors.

Visceral fat mass was compared across groups using either one-way ANOVA or the Kruskal–Wallis test, as appropriate.

A significance threshold of *p* < 0.05 was applied in all analyses. Statistical analyses were performed using IBM SPSS Statistics, version 25.

## 3. Results

### 3.1. Energy Intake

#### 3.1.1. Cumulative Energy Intake of Female Mice Groups

We first present the cumulative energy intake of the female mice groups kept on different diets during the bacterial administration period. The ND + CM group (545.03 ± 11.33 kcal) exhibited a significantly higher cumulative energy intake compared to the ND group (408.36 ± 72.67 kcal; *p* = 0.01). Conversely, the ND group (408.36 ± 72.67 kcal) demonstrated a significantly lower energy intake relative to the SD group (545.43 ± 5.52 kcal; *p* = 0.004). Furthermore, the HFD + CM group (388.53 ± 25.05 kcal) showed a significantly lower energy intake than the ND + CM group (545.03 ± 11.33 kcal; *p* < 0.001).

**Figure 1:**
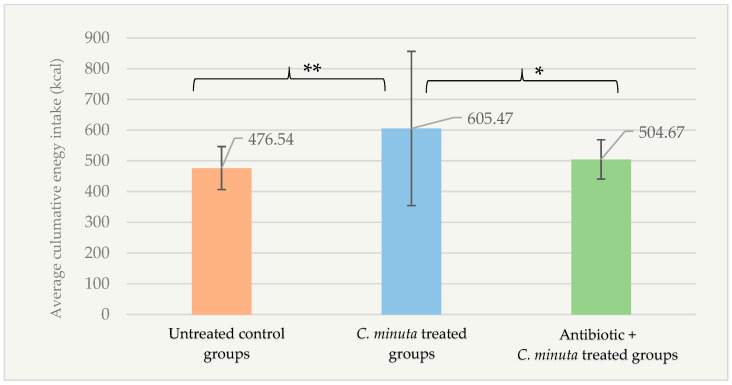
Average cumulative energy intake (kcal) of female mice treatment groups during the bacterial administration phase of the study (*n* = 89). * *p* < 0.05; ** *p* < 0.01.

According to the factorial design, the mean cumulative energy intake of the *C. minuta* treated groups (605.47 ± 251.20 kcal) was significantly higher than that of the untreated control groups (476.54 ± 69.99 kcal; *p* = 0.005) and the antibiotic + *C. minuta* treated groups (504.67 ± 63.89 kcal; *p* = 0.041) (Figure 1).

During the follow-up phase, the ND + CM group (632.75 ± 23.12 kcal) exhibited a significantly higher cumulative energy intake compared to the ND group (534.02 ± 37.78 kcal; *p* = 0.01). Additionally, the energy intake of the ND + CM group was significantly higher than that of the ND + AB + CM group (525.51 ± 9.35 kcal; *p* = 0.047). The HFD + CM group demonstrated a significantly lower energy intake (460.20 ± 22.71 kcal) compared to the ND + CM group (632.75 ± 23.12 kcal; *p* < 0.001).

According to the factorial design analysis, no significant differences in energy intake were observed among the treatment groups during the three-week bacterial administration period (*p* > 0.05).

#### 3.1.2. Cumulative Energy Intake of Male Mice Groups

In male mice groups, no significant differences in cumulative energy intake were observed during the bacterial administration period among any of the compared groups (*p* > 0.05). According to the factorial design analysis, no significant differences in energy intake were detected among the treatment groups during the three-week bacterial administration phase (*p* > 0.05).

#### 3.1.3. Cumulative Energy Intake of Male Mice Groups During the Follow up Period

The HFD + CM group (558.16 ± 17.16 kcal) exhibited a significantly lower energy intake compared to the ND + CM group (685.80 ± 11.39 kcal; *p* = 0.010). This finding is similar to that observed in female mice treated under the same conditions. The ND group (683.22 ± 0.51 kcal) demonstrated a significantly lower energy intake than the SD group (816.08 ± 78.08 kcal; *p* = 0.047).

**Figure 2:**
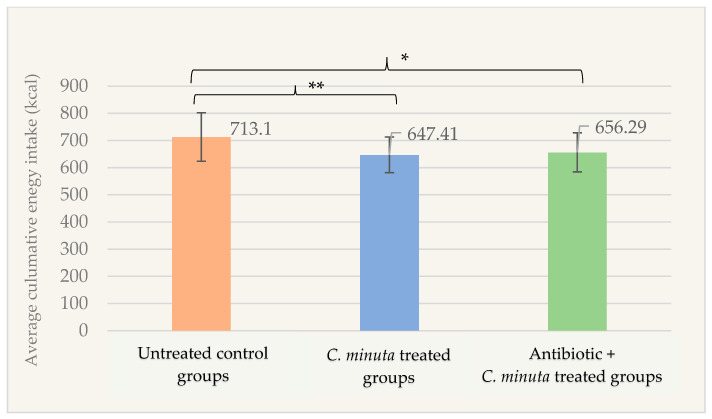
Average cumulative energy intake (kcal) of male mice treatment groups during the bacterial administration phase of the study (*n* = 89). * *p* < 0.05; ** *p* < 0.01.

Based on the factorial design, the mean cumulative energy intake measured during the follow-up period was significantly higher in the untreated control groups (713.10 ± 89.42 kcal) compared to the *C. minuta* treated groups (647.41 ± 65.46 kcal; *p* = 0.004) and the antibiotic treated + *C. minuta* treated groups (656.29 ± 71.90 kcal; *p* = 0.015) (Figure 2).

### 3.2. Results on the Impact of C. minuta on Body Weight

#### 3.2.1. Alterations in Body Weight Among Female Mice Groups

**Figure 3:**
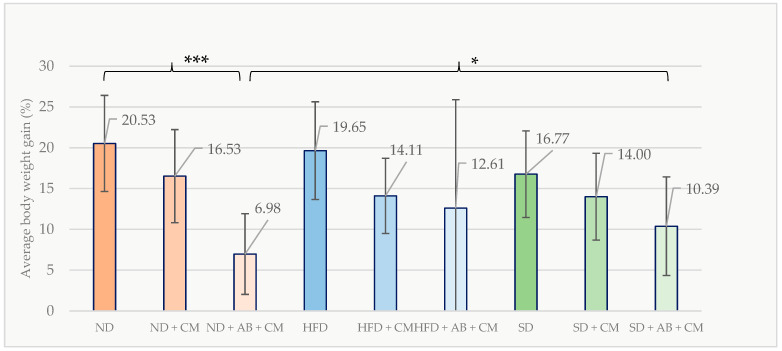
Percentage of body weight gain in female mice groups after *C. minuta* administration (*n* = 89). * *p* < 0.05; *** *p* < 0.001.

Alterations in body weight among the female groups were also assessed during the post-discontinuation period of bacterial administration. In the ND group, there was 20.53 ± 5.89% increase in body weight during this period, whereas the ND + AB + CM group demonstrated a significantly reduced increase of 6.98 ± 4.94% (*p* = 0.001). The body weight gain in the ND + AB + CM group (6.98 ± 4.94%) was also significantly lower compared to the SD + AB + CM group (10.39 ± 6.58%; *p* = 0.017). Following the active *C. minuta* administration period, body weight gain percentages were compiled across all female groups (Figure 3).

**Figure 4:**
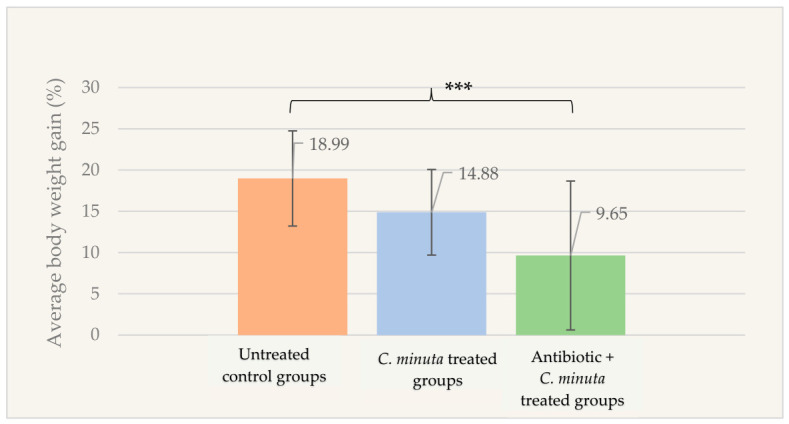
Percentage of body weight gain in female mice groups after *C. minuta* administration (*n* = 89). *** *p* < 0.001.

As defined by the factorial design, body weight gain was significantly lower in the antibiotic and *C. minuta* treated groups (9.65 ± 9.02%) compared to the untreated control groups (18.99 ± 5.77%; *p* < 0.001) (Figure 4).

The percentage of body weight gain was independent of energy intake (*p* > 0.05).

In the female mice groups, no significant differences in body weight gain were observed among any of the groups by the end of the experiment (*p* > 0.05).

**Figure 5:**
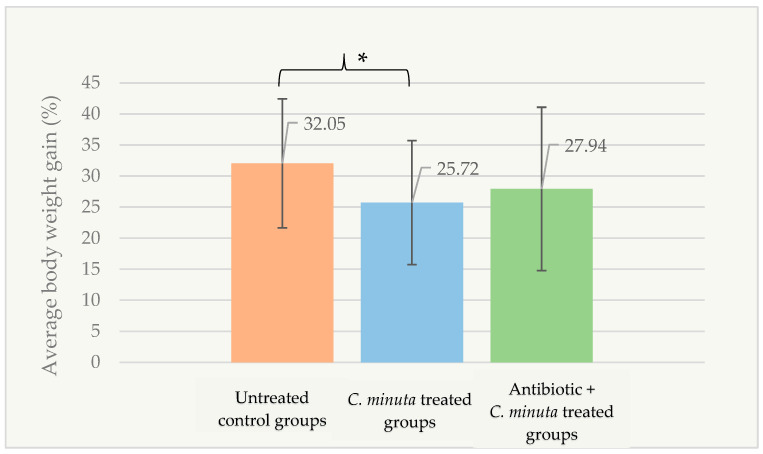
Percentage of body weight gain in female groups at the end of the experiment according to the treatment groups (*n* = 89). * *p* = 0.05.

Based on the factorial design, the *C. minuta* treated groups exhibited significantly lower body weight gain (25.72 ± 9.99%) compared to the untreated control groups (32.05 ± 10.38%), reaching the threshold for statistical significance (*p* = 0.05) (Figure 5). Body weight gain was independent of energy intake (*p* > 0.05).

#### 3.2.2. Alterations in Body Weight Among Male Mice Groups

**Figure 6:**
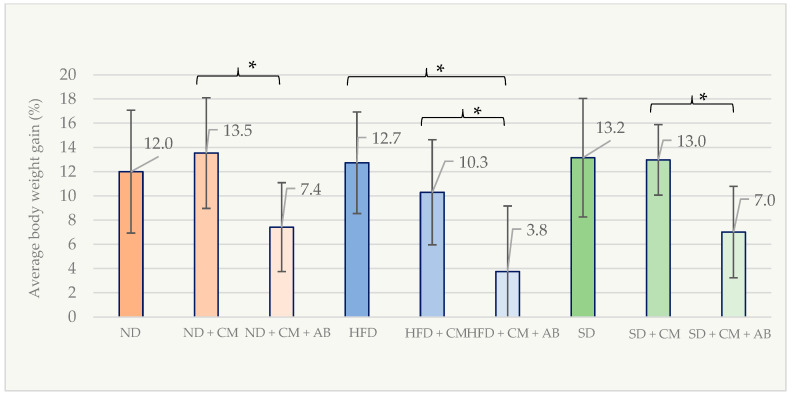
Percentage of body weight gain in male mice groups after *C. minuta* administration (*n* = 88). * *p* < 0.05.

Alterations in body weight among male mice groups were also measured during the post-discontinuation period of bacterial administration. Body weight gain in the ND + AB + CM group (7.42 ± 3.67%) was significantly lower than that in the ND + CM group (13.54 ± 4.57%; *p* = 0.024). The HFD + AB + CM group exhibited significantly lower body weight gain (3.75 ± 5.42%) compared to both the HFD group (12.74 ± 4.19%; *p* = 0.001) and the HFD + CM group (10.30 ± 4.34%; *p* = 0.045). The percentage of body weight gain observed in the SD + AB + CM group (6.62 ± 3.77%) was also significantly lower than that of the SD group (13.16 ± 4.89%; *p* = 0.046). A summary of the percentage of body weight gain in male animals following bacterial treatment is presented in Figure 6.

**Figure 7:**
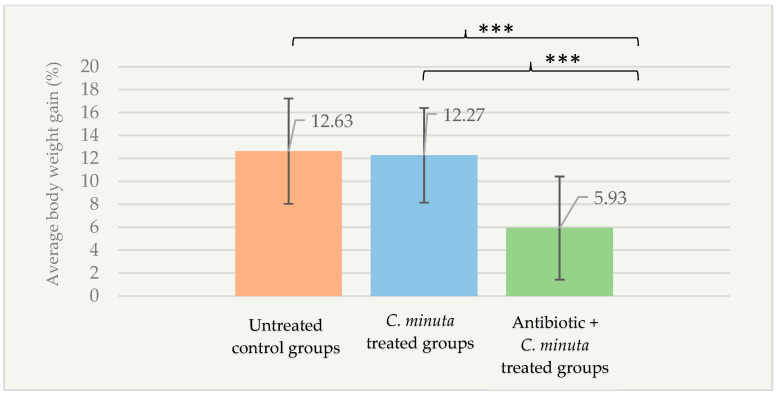
Percentage of body weight gain in male mice groups following *C. minuta* administration according to the treatment groups (*n* = 88). *** *p* < 0.001.

According to the factorial design, the body weight gain percentage of the antibiotic + *C. minuta* treated groups (5.93 ± 4.50%) was significantly lower than that of the untreated control groups (12.63 ± 4.59%; *p* < 0.001) and the *C. minuta* treated groups (12.27 ± 4.13%; *p* < 0.001) (Figure 7). The percentage of body weight gain was independent of energy intake (*p* > 0.05).

**Figure 8:**
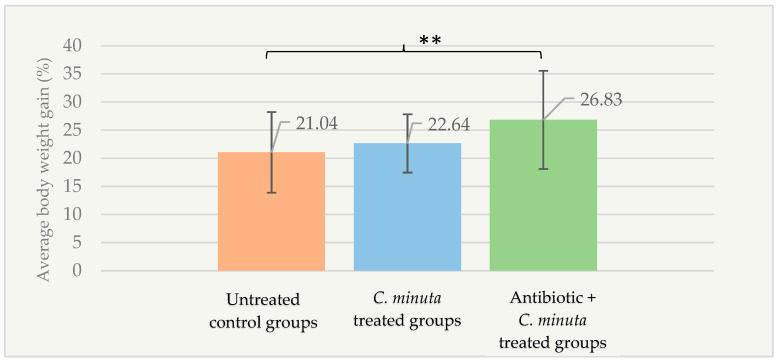
Percentage of body weight gain in male mice groups at the conclusion of the experiment according to the treatment groups (*n* = 88). ** *p* < 0.01.

Changes in body weight of male groups at the conclusion of the experiment were analyzed, and the nonparametric Kruskal–Wallis test revealed no statistically significant differences among the groups (*p* < 0.05). Based on the factorial design, body weight gain measured at the conclusion of the experiment showed a pattern contrary to that observed following bacterial treatment: in the antibiotic + *C. minuta* treated groups, body weight gain reached 26.83 ± 8.73%, which was significantly higher than in the untreated control groups (21.04 ± 7.17%; *p* = 0.009) (Figure 8).

The percentage of body weight gain was independent of energy intake (*p* > 0.05).

### 3.3. Results of Visceral Fat Measurements

#### 3.3.1. Comparison of Visceral Fat Mass in Female Mice Groups

Among the high-fat diet groups, significantly lower visceral fat mass was measured in the HFD + CM group (502.51 ± 245.29 mg) compared to the HFD group (1535.72 ± 891.71 mg; *p* = 0.004). Mice in the SD group exhibited significantly greater visceral fat mass (1403.60 ± 1103.76 mg) than those in the SD + CM group (276.39 ± 282.92 mg; *p* = 0.004) and SD + CM + AB group (387.22 ± 236.29 mg; *p* = 0.024).

**Figure 9:**
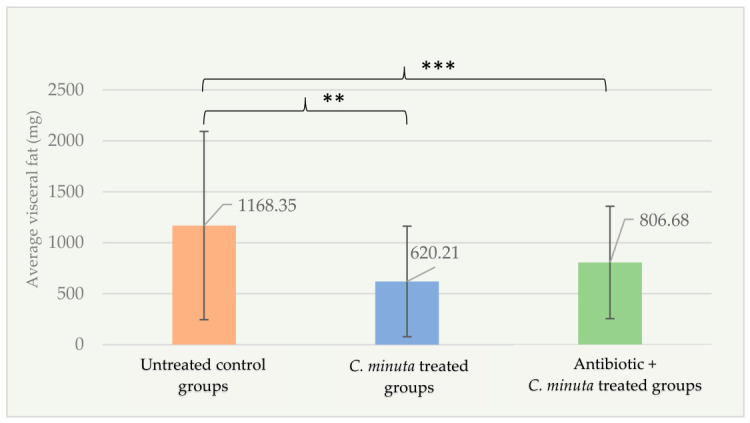
Visceral fat mass (mg) in female mice groups (*n* = 89). ** *p* < 0.01; *** *p* < 0.001.

According to the factorial design, visceral fat mass in the untreated groups (1168.35 ± 923.75 mg) was significantly higher compared to both the *C. minuta* treated groups (620.22 ± 542.28 mg; *p* < 0.001) and the antibiotic + *C. minuta* treated groups (806.68 ± 550.88 mg; *p* = 0.01) (Figure 9).

#### 3.3.2. Comparison of Visceral Fat Mass in Male Mice Groups

In male mice the visceral fat mass recorded in the ND + AB + CM group (1242.45 ± 506.23 mg) was significantly greater than that in the SD + AB + CM group (396.11 ± 315.77 mg; *p* = 0.004). No significant differences in visceral fat mass were observed among the male mice groups based on treatment allocation (*p* > 0.05).

## 4. Discussion

This study investigated the effects of *Christensenella minuta* administration on body weight gain under varying dietary conditions in male and female mice, with special attention to the role of energy intake and temporal dynamics of bacterial influence.

A novelty of the present study lies in its integrated experimental design, in which the effects of *Christensenella minuta* were evaluated across multiple dietary contexts (standard diet, high-fat diet, and sweetener exposure), both with and without antibiotic pretreatment, and in both male and female animals. In addition, both the intervention phase and the post-treatment period were assessed, allowing the evaluation of short-term effects and their persistence after cessation of administration. The intrarectal delivery approach further distinguishes this work from previous studies that have predominantly used oral administration.

A strength of our study is the inclusion of both male and female animals, as increasing evidence suggests that sex, as a biological variable, plays a significant role in host–microbiome interactions as well as in metabolic regulation. This approach enhances the physiological relevance and generalizability of our findings. In our study, we observed sex-related differences, the underlying mechanisms of which warrant further investigation. As this study was primarily designed as a proof-of-concept investigation, further work exploring different doses and routes of administration may provide additional mechanistic insight.

The results demonstrated significant reductions in weight gain during the active supplementation period, particularly in the groups receiving both *C. minuta* and antibiotics. However, the lack of sustained effects post administration underscores the transient nature of these benefits and highlights the necessity of continuous supplementation for prolonged efficiency. Our findings support the notion that *Christensenella minuta* functions as an ecologically active bacterium capable of modulating host metabolism [14,21], particularly in scenarios characterized by diminished microbial diversity after antibiotic pretreatment [19]. The observed attenuation of weight gain during *C. minuta* administration aligns with existing evidence of its role in shaping gut microbial communities via cross-feeding, nutrient competition, and metabolic complementation [21].

Nevertheless, the transient nature of this effect observed in our study indicates that *C. minuta* may necessitate sustained colonization or continuous administration to achieve its long-term metabolic benefits. Therefore, while it shows promise as a microbial intervention, its durability and therapeutic potential are likely contingent upon host-microbiota compatibility and ecological stability within the gut [21].

During the three-week administration period, mice treated with *C. minuta* exhibited a significantly reduced body weight gain compared to the control group, irrespective of caloric intake. This effect was observed in both male and female mice, although sex specific differences emerged over an extended duration. Statistical analyses, including ANCOVA, confirmed that the observed reduction in weight gain was attributable to bacterial treatment rather than variations in energy consumption, suggesting that the effects of *C. minuta* are not explained by differences in energy intake at the phenotypic level, although the underlying mechanisms remain to be elucidated. These results are consistent with previous studies indicating that *C. minuta* can modulate metabolic parameters independently of dietary quantity [14,17].

Our results further refine the findings of Goodrich et al. [12], who observed a reduction in body weight gain following a single fecal microbiota transplantation enriched with *C. minuta*. While their findings pointed to a genetic-microbiome interaction, they did not assess sustained or repeated interventions in their study. In contrast, Mazier et al. [14] provided daily oral supplementation of *C. minuta* DSM33407 for three weeks and found significant reductions in fat mass and improved metabolic markers without affecting the caloric intake. Similar to their findings, our study also demonstrated that *C. minuta* supplementation leads to reduced body weight gain, even under high-fat diet conditions. However, unlike Mazier’s continuous administration model, our once weekly intrarectal delivery showed that the anti-obesogenic effects were mostly transient, emphasizing the importance of the administration frequency and duration. Moreover, the transient nature of the effect may also be explained by the ecological interactions between *C. minuta* and other microbial taxa. Recent studies suggest that *C. minuta* engages in metabolic cross-feeding, nutrient competition, and complementation of its own deficiencies, which can influence the survival and proliferation of both itself and other community members. These interactions may limit its long-term colonization or functional dominance in the absence of sustained selective pressure or repeated administration [17].

Another key observation is that caloric intake did not significantly predict body weight gain in either sex, at any time point, despite group-level differences in energy consumption. For instance, even when *C. minuta* -treated mice consumed more or less energy depending on the diet type, they still exhibited altered body weight trajectories, further supporting the hypothesis that *C. minuta*’s effects are mediated via mechanisms distinct from energy intake, such as microbial metabolite production, immune modulation, or alterations in nutrient absorption [12,14,17,18]. This is consistent with findings by Mazier et al. [14], who reported improved metabolic outcomes in *C. minuta* treated mice without alterations in food intake. Liu et al., who identified specific microbial bile acid transformations as a key pathway of *C. minuta*’s systemic metabolic influence [17].

Emerging evidence highlights several mechanistic pathways through which *C. minuta* modulates host metabolism. Liu et al. demonstrated that *C. minuta* transforms primary bile acids into unique 3-O-acylated secondary bile acids that inhibit intestinal FXR signaling, a process associated with improved lipid metabolism and reduced inflammation [17]. Additionally, the administration of *C. minuta* in its live form has been shown to increase physical activity, reduce feed efficiency, and enhance energy loss via feces. In particular, female mice exhibited increased resting metabolic rate, suggesting sex-specific effects on energy expenditure. The observed female-specific effects may reflect sex-dependent differences in host microbiome interactions and hormonal regulation, as both estrogens and androgens are known to influence immune function, gut microbial composition, and metabolic homeostasis [33,34,35]. Furthermore, live *C. minuta* increases microbial biomass while reducing microbial diversity, and animals with higher *C. minuta* abundance lose more dietary energy in their stool [18]. These data collectively support the role of *C. minuta* as a keystone species with disproportionate influence on host metabolic homeostasis.

Regarding the effects of the bacterium on visceral fat, our study found that *C. minuta*, both as a standalone treatment and in combination with antibiotic pretreatment, significantly attenuated visceral fat accumulation in female groups compared to untreated controls. This observation is consistent with the findings of Mazier et al., who demonstrated similar effects in male mice on a high-fat diet, further reinforcing the potential of this bacterium to inhibit visceral adipose tissue expansion [14]. One proposed mechanism for this effect is the production of short-chain fatty acids (SCFAs), which modulate lipogenesis and lipolysis, thereby regulating visceral fat levels [36]. Additionally, 3-O-acylated secondary bile acids have been shown to inhibit not only body weight gain but also visceral fat accumulation [17]; however, these specific pathways were not evaluated in the present study.

These findings contribute to the growing body of evidence positioning *Christensenella minuta* as a next generation probiotic candidate with the potential to transiently modulate host metabolism and adiposity [37,38]. However, the lack of sustained effect after treatment cessation underscores the need for continuous or repeated administration protocols if *C. minuta* is to be considered for long-term therapeutic use in obesity management. Further studies are warranted to explore the optimal dosing regimens, colonization stability, and potential combinatory effects with diet or prebiotics to prolong its beneficial outcomes.

### 4.1. Study Limitations

This study had several limitations that must be acknowledged. First, owing to budgetary constraints, we did not perform a detailed microbiome analysis to investigate the compositional changes induced by *C. minuta* in the gut microbiota. Such data could provide deeper insights into the mechanisms underlying the observed metabolic effects. Second, the study relied on an intrarectal administration method, which, while innovative, may not reflect practical therapeutic scenarios for human applications. Finally, the absence of data on potential sex-specific microbiome changes limits the generalizability of our findings to a broader context.

### 4.2. Future Research Directions

Future studies should prioritize comprehensive microbiome profiling to elucidate the mechanistic pathways underlying the effects of *C. minuta*. Longitudinal studies assessing the feasibility of sustained administration and its potential to induce durable alterations in the gut microbiota are needed. Exploration of alternative administration routes, such as encapsulated oral formulations, to enhance translational relevance. Investigation of sex-specific responses, given the observed differences in caloric intake and weight regulation between male and female mice.

## 5. Conclusions

In this study, we demonstrated that intrarectal administration of *Christensenella minuta* significantly mitigated body weight gain in both male and female mice during the active treatment period, irrespective of dietary composition or caloric intake.

The effects were most pronounced during the three week administration phase. During the subsequent eight week follow-up period, the reduced body weight gain persisted exclusively in female animals, and only in those that did not receive the antibiotic pretreatment. This suggests that the longer term metabolic effect of *C. minuta* is not only gender specific but also dependent on the presence of an intact gut microbiome. Importantly, energy intake did not show a significant correlation with body weight gain in any group, whereas *C. minuta* treatment consistently reduced weight gain under both normal and high fat diet conditions during the active treatment phase. This indicates that the bacterium modulates host energy balance through mechanisms independent of energy consumption and dietary type. Overall, our findings highlight the short term efficacy of *Christensenella minuta* in reducing body weight gain independently of caloric intake, while also emphasizing that the persistence of this effect after cessation of treatment is sex specific and contingent upon the absence of antibiotic pretreatment. These results underscore the importance of microbiome integrity and warrant further mechanistic investigations into the metabolic effects of *C. minuta*.

## Figures and Tables

**Table 1:** Description of the experimental groups based on diet type and treatment conditions.

Group	Diet	Treatment
1. ND	Standard diet	None
2. ND + CM	Standard diet	*C. minuta*
3. ND + CM + AB	Standard diet	Fluoroquinolone pretreatment + *C. minuta*
4. HFD	High fat diet	None
5. HFD + CM	High fat diet	*C. minuta*
6. HFD + CM + AB	High fat diet	Fluoroquinolone pretreatment + *C. minuta*
7. SD	Sweetener diet	None
8. SD + CM	Sweetener diet	*C. minuta*
9. SD + AB + CM	Sweetener diet	Fluoroquinolone pretreatment + *C. minuta*

## Data Availability

Data is available from the corresponding author upon reasonable request.

## Supplementary Materials

The following supporting information can be downloaded at: https://www.mdpi.com/article/10.3390/nu18101593/s1, Figure S1: “Ethics approval document”; Figure S2: “Rat/mouse maintainance feed”; Figure S3: “Mouse high fat pellet”; Figure S4: “Mean body weight (g) and body weight gain (%) of female and male groups over the entire study period”; Figure S5: “Mean body weight (g) and body weight gain (%) of female groups over the entire study period (*n* = 89)”; Figure S6: “Body weight changes (%) in male groups fed a normal diet (ND) (*n* = 30)”; Figure S7: “Body weight changes (%) in female HFD groups (*n* = 30)”; Figure S8: “Body weight changes (%) in female mice consuming sweetener-containing fluid (SD) (*n* = 29)”; Figure S9: “Mean body weight (g) and body weight gain (%) of male groups over the entire study period (*n* = 88)”; Figure S10: “Body weight changes (%) in male mice fed a normal diet (ND) (*n* = 30)”; Figure S11: “Body weight changes (%) in male mice fed a high-fat diet (HFD) (*n* = 28)”; Figure S12: “Body weight changes (%) in male mice consuming sweetener-containing fluid (SD) (*n* = 30)”; Figure S13: “Mean energy intake (kcal) of female and male groups over the entire study period”; Figure S14: “Cumulative mean energy intake (kcal) of female groups over the entire study period (*n* = 89)”; Figure S15: “Cumulative energy intake (kcal) in female groups fed a normal diet (ND) (*n* = 30)”; Figure S16: “Cumulative energy intake (kcal) in female groups fed a high-fat diet (HFD) (*n* = 30)”; Figure S17: “Cumulative energy intake (kcal) in female groups consuming sweetener-containing fluid (SD) (*n* = 29)”; Figure S18: “Cumulative mean energy intake (kcal) of male groups over the entire study period (*n* = 88)”; Figure S19: “Cumulative energy intake (kcal) in male groups fed a normal diet (ND) (*n* = 30)”; Figure S20: “Cumulative energy intake (kcal) in male groups fed a high-fat diet (HFD) (*n* = 28)”; Figure S21: “Cumulative energy intake (kcal) in male groups consuming sweetener-containing fluid (SD) (*n* = 30)”.
