# Supplementary material for: Does Intrarectal Administration of Christensenella minuta DSM22607 Impact Body Weight?
Source: Nutrients. 2026 May 17;18(10):1593. doi: 10.3390/nu18101593 (PMC13210250; doi:10.3390/nu18101593)
Supplement: Supplementary file 1 [file nutrients-18-01593-s001.zip › nutrients-4283853-supplementary.pdf]

Supplementary material

Does Intrarectal Administration of *Christensenella minuta* DSM22607 Impact Body Weight?

Dorottya Zsálig [1,*], Ádám Molnár [2], Monika Kerényi [3], Fruzsina Péter [4], Gellért Gerencsér [2,5] and Éva Polyák [4,*]

1. Supplementary Methods

Animal Housing and Conditions

This study was conducted using 180 six-week-old mice, sourced from the in-house breeding facility of the Preclinical Research Centre. The mice were housed in the Centre's animal facility, specifically in the designated mouse room. Each cage measured 425 × 266 × 150 mm, with separate housing for each sex, arranged in groups of five according to the experimental design. The room was maintained at a temperature of 22 ± 2 °C, with humidity levels between 45–60%, and a 12-hour light/dark cycle facilitated by artificial lighting. Dust-free wood shavings were utilized as bedding, and the cages were cleaned every 1–2 days. The room underwent daily cleaning and disinfection. Paper toys were provided for environmental enrichment. The condition of the animals was monitored daily. The animal facility was under regular veterinary supervision, with annual serological examinations conducted on the animals. Comprehensive records of the animals' housing, origin, and experimental involvement were maintained by the Preclinical Research Centre for a minimum of five years.

In vivo Methodology

Study Design

The study was granted approval by the Baranya County Government Office, Department of Food Chain Safety and Animal Health, which functions as the animal welfare authority (BA02/2000-21/2024).

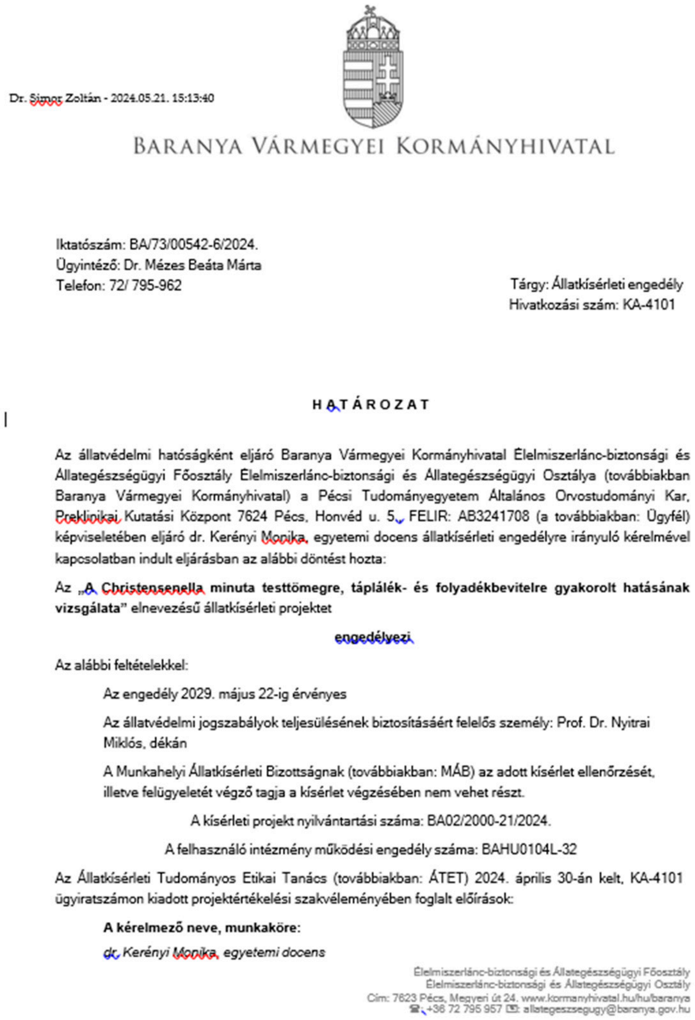

Dr. Simor Zoltán - 2024.05.21. 15:13:40

BARANYA VÁRMEGYEI KORMÁNYHIVATAL

Iktatószám: BA/73/00542-6/2024.
Ügyintéző: Dr. Mézes Beáta Márta
Telefon: 72/ 795-962

Tárgy: Állatkísérleti engedély
Hivatkozási szám: KA-4101

HATÁROZAT

Az állatvédelmi hatóságként eljáró Baranya Vármegyei Kormányhivatal Élelmiszerlánc-biztonsági és Állategészségügyi Főosztály Élelmiszerlánc-biztonsági és Állategészségügyi Osztálya (továbbiakban Baranya Vármegyei Kormányhivatal) a Pécsi Tudományegyetem Általános Orvostudományi Kar, Preklinikai Kutatási Központ 7624 Pécs, Honvéd u. 5. FELIR: AB3241708 (a továbbiakban: Ügyfél) képviseletében eljáró dr. Kerényi Monika, egyetemi docens állatkísérleti engedélyre irányuló kérelmével kapcsolatban indult eljárásban az alábbi döntést hozta:

Az **„A Christensenella minuta testtömegre, táplálék- és folyadékbevitelre gyakorolt hatásának vizsgálata"** elnevezésű állatkísérleti projektet

engedélyezi

Az alábbi feltételekkel:

Az engedély 2029. május 22-ig érvényes

Az állatvédelmi jogszabályok teljesülésének biztosításáért felelős személy: Prof. Dr. Nyitrai Miklós, dékán

A Munkahelyi Állatkísérleti Bizottságnak (továbbiakban: MÁB) az adott kísérlet ellenőrzését, illetve felügyeletét végző tagja a kísérlet végzésében nem vehet részt.

A kísérleti projekt nyilvántartási száma: BA02/2000-21/2024.

A felhasználó intézmény működési engedély száma: BAHU0104L-32

Az Állatkísérleti Tudományos Etikai Tanács (továbbiakban: ÁTET) 2024. április 30-án kelt, KA-4101 ügyiratszámon kiadott projektértékelési szakvéleményében foglalt előírások:

A kérelmező neve, munkaköre:

dr. Kerényi Monika, egyetemi docens

Élelmiszerlánc-biztonsági és Állategészségügyi Főosztály
Élelmiszerlánc-biztonsági és Állategészségügyi Osztály
Cím: 7623 Pécs, Megyeri út 24. www.kormanyhivatal.hu/hu/baranya
☎: +36 72 795 957 ✉: allategeszsegugy@baranya.gov.hu

Figure S1. Ethics approval document.

The animal experiment was conducted at the Preclinical Research Center, Faculty of Medicine, University of Pécs. Six-week-old male and female CD1 inbred mice (Mus musculus) were utilized. The animals were housed in a conventional animal facility under standard conditions (22 ± 2 °C, 45–60% relative humidity) with a 12-hour light/dark cycle using artificial lighting that corresponded to the natural photoperiod. Bedding consisted of dust-free wood shavings, and cages were cleaned every 1–2 days. A total of five animals per cage were housed. The experiment spanned 12 weeks and comprised three main phases: an acclimatization and antibiotic pretreatment phase (week 1), a 3-week *C. minuta* administration period (weeks 2–4), and an 8-week long-term follow-up phase (weeks 5–11) during which animals remained on the same diets to assess sustained effects. In week 12, final body weight was measured, followed by euthanasia. Group allocation involved a total of 180 six-week-old CD1 mice (90 males and 90 females), with equal sex distribution. Animals were randomly assigned to nine experimental groups, with 20 animals per group (10 females, 10 males). Below, animals are described according to diet and treatment.

1.1 Control group (ND): The animals were provided with a standard chow diet, comprising 9% fat, 24% protein, and 67% carbohydrates, totaling 3225 kcal/kg, with 5 g of fiber per 100 g, and had unrestricted access to tap water. No additional treatment was

administered. In this study, the male and female cohorts were designated as ND (normal diet).

1.2 Normal diet + *C. minuta* treatment group (ND + CM): The animals were maintained on the same diet as the control group and were additionally administered a *C. minuta* suspension intrarectally on three separate occasions. The male and female cohorts were designated as ND + CM (normal diet + *C. minuta*).

1.3 Normal diet + antibiotic pretreatment + *C. minuta* treatment group (ND + AB + CM): The animals received antibiotics in their drinking water for one week prior to the commencement of the experiment. The male and female cohorts were designated as ND + AB + CM (normal diet + antibiotic + *C. minuta*). The animals were maintained on the same diet as the control group (ND) and were additionally administered a *C. minuta* suspension intrarectally.

2.1 High-fat diet group (HFD): The animals consumed a high-fat diet, consisting of 18% fat, 24% protein, and 58% carbohydrates, totaling 3100 kcal/kg, with 3.8 g of fiber per 100 g, and had unrestricted access to tap water. The male and female cohorts were designated as HFD (high-fat diet).

2.2 High-fat diet + *C. minuta* treatment group (HFD + CM): The animals were maintained on the same high-fat diet as the HFD group and were additionally administered a *C. minuta* suspension intrarectally on three separate occasions. The male and female cohorts were designated as HFD + CM (high-fat diet + *C. minuta*).

2.3 High-fat diet + antibiotic pretreatment + *C. minuta* treatment group (HFD + AB + CM): The animals received antibiotics in their drinking water for one week prior to the commencement of the experiment. The animals were maintained on the same high-fat diet as the HFD group and were additionally administered a *C. minuta* suspension intrarectally. The male and female cohorts were designated as HFD + AB + CM (high-fat diet + antibiotic + *C. minuta*).

3.1. Sweetener-Fluid Group (SD): In this study, the animals were provided with water containing sweetener (5 Süssina tablets per 300 mL of water) and standard chow, both available ad libitum. The male and female cohorts were designated as SD (sweetener diet).

3.2. Sweetener-Fluid Plus *C. minuta* Treatment Group (SD + CM): This group received the same diet and fluid as the SD group, with the addition of an intrarectal suspension of *C. minuta*. The male and female cohorts were designated as SD + CM (sweetener diet plus *C. minuta*).

3.3. Sweetener-Fluid Plus Antibiotic Pretreatment Plus *C. minuta* Treatment Group (SD + AB + CM): Prior to the commencement of the experiment, these animals were administered antibiotics in their drinking water for one week. They were then provided with the same diet and sweetener-containing fluid as the SD group, along with an intrarectal suspension of *C. minuta*. The male and female cohorts were designated as SD + AB + CM (sweetener diet plus antibiotic plus *C. minuta*).

Based on dietary intervention, the groups were categorized as follows: normal diet groups (ND, ND + CM, ND + AB + CM), high-fat diet groups (HFD, HFD + CM, HFD + AB + CM), and standard diet groups receiving sweetener-containing fluids (SD, SD + CM, SD + AB + CM). Based on treatment, the groups were categorized as "no-treatment groups" (ND, HFD, SD), "*C. minuta*-treated groups" (ND + CM, HFD + CM, SD + CM), and "antibiotic plus *C. minuta*-treated groups" (ND + AB + CM, HFD + AB + CM, SD + AB + CM).

Characteristics of Feeding

In the conventional chow groups (ND, ND + CM, ND + AB + CM) and the sweetener-supplemented groups (SD, SD + CM, SD + AB + CM), the animals were maintained on a

standard diet comprising 9% fat, 24% protein, and 67% carbohydrates, with an energy content of 3225 kcal/kg and 5 g fiber/100 g (Ssniff, Rat/mouse maintenance).

Detailed dietary composition is provided in Figure S2.

In contrast, the high-fat diet groups (HFD, HFD + CM, HFD + AB + CM) received a diet where 18% of the energy was derived from fat, 24% from protein, and 58% from carbohydrates, with an energy content of 3100 kcal/kg and 3.8 g fiber/100 g (Teklad Global Rodent Diet (Sterilizable) 2018S). Detailed dietary composition is provided in Figure S3.

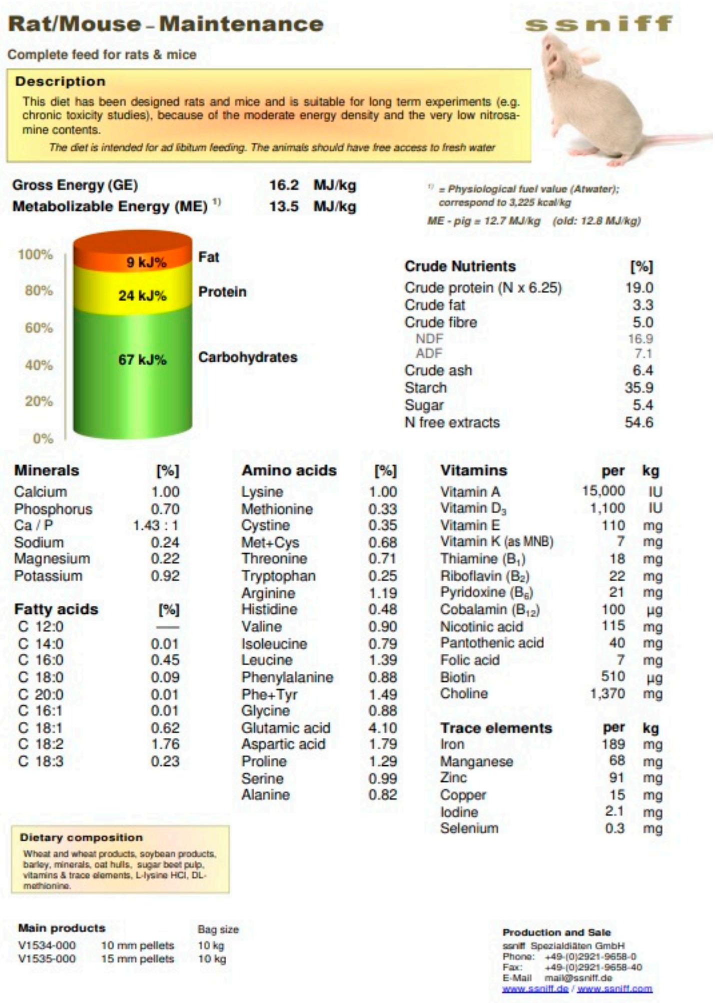

Rat/Mouse - Maintenance

ssniff

Complete feed for rats & mice

Description

This diet has been designed rats and mice and is suitable for long term experiments (e.g. chronic toxicity studies), because of the moderate energy density and the very low nitrosamine contents.

The diet is intended for ad libitum feeding. The animals should have free access to fresh water

Gross Energy (GE)	16.2	MJ/kg
Metabolizable Energy (ME) [1)]	13.5	MJ/kg

[1)] = Physiological fuel value (Atwater); correspond to 3,225 kcal/kg

ME - pig = 12.7 MJ/kg (old: 12.8 MJ/kg)

Crude Nutrients	[%]
Crude protein (N x 6.25)	19.0
Crude fat	3.3
Crude fibre	5.0
NDF	16.9
ADF	7.1
Crude ash	6.4
Starch	35.9
Sugar	5.4
N free extracts	54.6

Minerals	[%]
Calcium	1.00
Phosphorus	0.70
Ca / P	1.43 : 1
Sodium	0.24
Magnesium	0.22
Potassium	0.92

Fatty acids	[%]
C 12:0	—
C 14:0	0.01
C 16:0	0.45
C 18:0	0.09
C 20:0	0.01
C 16:1	0.01
C 18:1	0.62
C 18:2	1.76
C 18:3	0.23

Amino acids	[%]
Lysine	1.00
Methionine	0.33
Cystine	0.35
Met+Cys	0.68
Threonine	0.71
Tryptophan	0.25
Arginine	1.19
Histidine	0.48
Valine	0.90
Isoleucine	0.79
Leucine	1.39
Phenylalanine	0.88
Phe+Tyr	1.49
Glycine	0.88
Glutamic acid	4.10
Aspartic acid	1.79
Proline	1.29
Serine	0.99
Alanine	0.82

Vitamins	per	kg
Vitamin A	15,000	IU
Vitamin D_3	1,100	IU
Vitamin E	110	mg
Vitamin K (as MNB)	7	mg
Thiamine (B_1)	18	mg
Riboflavin (B_2)	22	mg
Pyridoxine (B_6)	21	mg
Cobalamin (B_{12})	100	µg
Nicotinic acid	115	mg
Pantothenic acid	40	mg
Folic acid	7	mg
Biotin	510	µg
Choline	1,370	mg

Trace elements	per	kg
Iron	189	mg
Manganese	68	mg
Zinc	91	mg
Copper	15	mg
Iodine	2.1	mg
Selenium	0.3	mg

Dietary composition

Wheat and wheat products, soybean products, barley, minerals, oat hulls, sugar beet pulp, vitamins & trace elements, L-lysine HCl, DL-methionine.

Main products		Bag size
V1534-000	10 mm pellets	10 kg
V1535-000	15 mm pellets	10 kg

Production and Sale

ssniff Spezialdiäten GmbH
Phone: +49-(0)2921-9658-0
Fax: +49-(0)2921-9658-40
E-Mail mail@ssniff.de
www.ssniff.de / www.ssniff.com

Figure S2. Rat/mouse maintainance feed.

2018S/2018SC

Teklad Global 18% Protein Rodent Diet (Sterilizable)

inotiv
analyze. answer. advance.

Product Description - 2018S is a fixed formula, autoclavable diet manufactured with high quality ingredients designed to support gestation, lactation, and growth of rodents. 2018S exludes alfalfa meal, which lowers phytoestrogen (coumestrol) content, and reduces chlorophyll, improving optical imaging clarity. A moderate inclusion of soybean meal results in an expected isoflavone range of 225-340 mg/kg diet (daidzein + genistein aglycone equivalents). Absence of fish meal minimizes the presence of nitrosamines. 2018S is supplemented with additional vitamins to ensure nutritional adequacy after autoclaving. **Related codes 2018, 2018C (certified), 2918C (irradiated, certified), 2018X (extruded), 2918X (irradiated, extruded); autoclavable version 2018SX (sterilizable, extruded). Most commonly sold as 2018SC (certified) in North America.**

Ingredients (in descending order of inclusion)- Ground wheat, ground corn, wheat middlings, dehulled soybean meal, corn gluten meal, soybean oil, calcium carbonate, dicalcium phosphate, brewers dried yeast, iodized salt, L-lysine, DL-methionine, choline chloride, menadione sodium bisulfite complex (source of vitamin K activity), magnesium oxide, vitamin E acetate, calcium pantothenate, thiamin mononitrate, manganous oxide, niacin, ferrous sulfate, zinc oxide, riboflavin, vitamin A acetate, pyridoxine hydrochloride, copper sulfate, vitamin B_{12} supplement, folic acid, calcium iodate, biotin, vitamin D_3 supplement, cobalt carbonate.

Standard Product Form: ***Pellet***

Macronutrients		
Crude Protein	%	18.4
Fat (ether extract) [a]	%	6.0
Carbohydrate (available) [b]	%	44.2
Crude Fiber	%	3.8
Neutral Detergent Fiber [c]	%	14.7
Ash	%	5.5
Energy Density [d]	kcal/g (kJ/g)	3.1 (13.0)
Calories from Protein	%	24
Calories from Fat	%	18
Calories from Carbohydrate	%	58
**Minerals**		
Calcium	%	1.0
Phosphorus	%	0.7
Non-Phytate Phosphorus	%	0.4
Sodium	%	0.2
Potassium	%	0.6
Chloride	%	0.4
Magnesium	%	0.2
Zinc	mg/kg	70
Manganese	mg/kg	100
Copper	mg/kg	15
Iodine	mg/kg	6
Iron	mg/kg	200
Selenium	mg/kg	0.23
**Amino Acids**		
Aspartic Acid	%	1.4
Glutamic Acid	%	3.4
Alanine	%	1.1
Glycine	%	0.8
Threonine	%	0.7
Proline	%	1.6
Serine	%	1.1
Leucine	%	1.8
Isoleucine	%	0.8
Valine	%	0.9
Phenylalanine	%	1.0
Tyrosine	%	0.6
Methionine	%	0.6
Cystine	%	0.3
Lysine	%	1.1
Histidine	%	0.4
Arginine	%	1.0
Tryptophan	%	0.2

Vitamins		
Vitamin A [e, f]	IU/g	30.0
Vitamin D_3 [e, g]	IU/g	2.0
Vitamin E	IU/kg	135
Vitamin K_3 (menadione)	mg/kg	100
Vitamin B_1 (thiamin)	mg/kg	117
Vitamin B_2 (riboflavin)	mg/kg	27
Niacin (nicotinic acid)	mg/kg	115
Vitamin B_6 (pyridoxine)	mg/kg	26
Pantothenic Acid	mg/kg	140
Vitamin B_{12} (cyanocobalamin)	mg/kg	0.15
Biotin	mg/kg	0.90
Folate	mg/kg	9
Choline	mg/kg	1200
**Fatty Acids**		
C16:0 Palmitic	%	0.7
C18:0 Stearic	%	0.2
C18:1ω9 Oleic	%	1.2
C18:2ω6 Linoleic	%	3.1
C18:3ω3 Linolenic	%	0.3
Total Saturated	%	0.9
Total Monounsaturated	%	1.3
Total Polyunsaturated	%	3.4
**Other**		
Cholesterol	mg/kg	--

Shelf life: With proper storage, diet is suitable for use out to 9 months.

www.inotivco.com/shelf-life-of-diets-used-in-research

[a] Ether extract is used to measure fat in pelleted diets, while an acid hydrolysis method is required to recover fat in extruded diets. Compared to ether extract, the fat value for acid hydrolysis will be approximately 1% point higher.

[b] Carbohydrate (available) is calculated by subtracting neutral detergent fiber from total carbohydrates.

[c] Neutral detergent fiber is an estimate of insoluble fiber, including cellulose, hemicellulose, and lignin. Crude fiber methodology underestimates total fiber.

[d] Energy density is a calculated estimate of *metabolizable energy* based on the Atwater factors assigning 4 kcal/g to protein, 9 kcal/g to fat, and 4 kcal/g to available carbohydrate.

[e] Indicates added amount but does not account for contribution from other ingredients.

[f] 1 IU vitamin A = 0.3 μg retinol

[g] 1 IU vitamin D = 25 ng cholecalciferol

For nutrients not listed, insufficient data is available to quantify.

Nutrient data represent the best information available, calculated from published values and direct analytical testing of raw materials and finished product. Nutrient values may vary due to the natural variations in the ingredients, analysis, and effects of processing.

Teklad Diets are designed and manufactured for research purposes only.

SGS

Figure S3. Mouse high fat pellet.

Sweetener Administration

In alignment with the protocols applied to other experimental cohorts, the groups treated with sweeteners were provided with fresh drinking water biweekly, each time in a volume of 300 mL. In each instance, five commercially available S. sweetener tablets were dissolved in the water, with these tablets collectively containing 173.5 mg of sodium cyclamate and 46.5 mg of sodium saccharinate. Our methodology employed a concentration of 0.005%, which is significantly lower than the commonly reported range of 0.1–0.3% in existing literature. This methodological choice was intended to simulate a continuous, low-level, and safe intake, thereby reflecting realistic conditions of sugar substitution. The solutions were freshly prepared at consistent concentrations for each administration.

The consumption of sweetener-containing fluids commenced following a week of acclimatization, coinciding with the initiation of *C. minuta* administration across all sweetener-fluid groups. Our objective was to replicate the use of sweeteners frequently incorporated into weight-loss diets under controlled experimental conditions. Given the prevalent use of sweeteners among individuals pursuing weight loss and health-conscious populations, we investigated potential interactions between bacterial administration and sweetener consumption. The selection of commercial tabletop sweeteners was driven by their affordability and accessibility, making them a popular choice. In this study, sweetener administration was facilitated through drinking water, which may plausibly enhance fluid intake due to the preference for a sweet taste.

Antibiotic Pretreatment of Antibiotic Groups

In vivo antibiotic pretreatment, particularly in mouse models, serves as an experimental strategy to partially deplete the gut microbiota, thereby facilitating the colonization of newly introduced microorganisms, such as probiotics, pathogens, or donor microbiota, within the intestinal tract. This approach aims to enhance the efficacy of these microorganisms. In animal studies, short-term antibiotic administration does not result in increased body fat or body weight; however, prolonged antibiotic exposure may lead to persistent alterations in the microbiome, potentially resulting in increased body fat and/or body weight. These factors must be considered when interpreting study outcomes. During the acclimatization period, mice in the antibiotic-treated groups were provided with drinking water containing levofloxacin, a fluoroquinolone, at a concentration of 0.1 mg/mL (300 mL/day). This dosage is both clinically and microbiologically adequate to significantly reduce the gut microbiota. The antibiotic pretreatment was administered for 5 days, followed by 2 days of tap water, as previously described. Notably, no diarrhea was observed in the animals during the treatment period.

Intrarectal Administration of the Bacterium

Following a 12-hour fasting period, the groups treated with *C. minuta* underwent intrarectal bacterial administration once weekly for three consecutive weeks (weeks 2, 3, and 4), totaling three administrations. The bacterial suspension concentration was 10^9 CFU/mL, with 0.2 mL administered via a venous catheter (B. Braun, Hungary, Certofix Mono V 415 1, 2 V, G18×70 mm) at room temperature. The catheter was inserted in a standardized manner in all animals to a depth of at least 2.5 cm into the lumen of the distal colon, very slowly to prevent injury. Our objective with this method was to integrate prior study experience by combining a repeated, intermittent dosing protocol with a fecal-transplantation-like intrarectal delivery route, directly targeting the colonic microbial community rather than employing an oral route. By selecting this route of administration, we aimed to circumvent upper gastrointestinal degradation and microbial competition associated with oral colonization, thereby facilitating a more precise evaluation of the direct mechanisms of action of *C. minuta*.

Measurements Performed During the Experiment

Body weight was measured three times per week using an analytical balance (Boeco BAS 31 Plus, Germany; accuracy: ±0.01 g). Each measurement was repeated three times consecutively, and the mean of these three measurements was used as the final value. A predetermined quantity of chow (300 g) was provided to the mice, and the remaining food in the feeder was weighed and subtracted from the initial chow mass. The difference was considered the amount consumed. Mean energy intake (kcal/g) was calculated based on the consumed chow mass and the energy content specified by the manufacturer. Standard chow contained 3225 kcal/kg (Manufacturer: Ssniff, Rat/mouse maintenance), whereas the high-fat diet contained 3100 kcal/kg (Manufacturer: Teklad Global Rodent Diet (Sterilizable) 2018S). Fluid intake was measured biweekly. A predetermined volume of tap water (300 mL) was provided. The remaining volume in the bottle was measured and subtracted from the initial volume, and the sum of the two differences was considered the amount of fluid consumed. Determinations of chow intake, fluid intake, and energy intake were performed cumulatively, i.e., per cage (5 animals per cage).

Termination and Sample Collection

During the experiment, three animals succumbed: two from the high-fat diet male group (HFD) and one from the sweetener + antibiotic + *C. minuta* female group (SD + AB + CM). Within the HFD group, one animal expired in the eighth week, while the other perished during the final week of the study. The female animal in the SD + AB + CM group died in the third week. The cause of death was attributed to aggressive behavior observed within the group, a phenomenon consistently noted in these groups. At the conclusion of the study, the remaining 177 animals were euthanized via cervical dislocation. During dissection, the kidneys, liver, and visible periorgan adipose tissues were excised and weighed (Boeco BAS 31 Plus, Germany; accuracy: ±0.01 g), and subsequently stored at −40 °C.

Statistical Analysis

The normality of data distribution for each variable was evaluated using the Shapiro–Wilk test. Differences between groups in body weight gain, chow/energy intake, and fluid intake were assessed using either one-way ANOVA or the Kruskal–Wallis test, contingent upon the normality of the distribution. For the assessment of body weight changes, the percentage of body weight gain was deemed the most appropriate metric due to baseline body weight (g) variations across groups. Consequently, body weight changes were analyzed in percentage terms to better control for confounding effects arising from baseline body weight differences. Correlation analysis was employed to assess associations between body weight and energy intake. A factorial experimental design was utilized to examine the effects and interactions of two independent variables: diet type (three levels: standard diet, high-fat diet, and standard diet with sweetener-containing fluid) and treatment type (three levels: no treatment, *C. minuta* treatment (CM), and *C. minuta* treatment following antibiotic pretreatment (AB + CM)). This 3 × 3 factorial design facilitated the evaluation of main effects (diet and treatment) and their interaction. Analyses were conducted separately for the active bacterial administration period and the long-term follow-up phase post-administration to evaluate both immediate and sustained effects. Body weight, energy intake, and fluid intake were analyzed as functions of the two independent factors. Statistical evaluation was performed using two-way ANOVA, separately for female and male animals, as significant biological sex differences—particularly in baseline body weight, metabolic profile, and responses to the bacterium—warranted sex-stratified data processing. Differences in body weight changes over the entire study period were assessed using repeated-measures ANOVA. Between-group differences in visceral fat mass were evaluated using one-way ANOVA or the Kruskal–Wallis test, depending on normality. For all analyses, a p-value of less than 0.05 was considered statistically significant. Statistical analyses were conducted using IBM SPSS Statistics version 25.

2. Supplementary Figures and Tables

Weight Gain Measurements

The weekly body weight measurements are summarized in the table below, presenting the weekly body weight (g), the increase in body weight compared to the previous week in grams, and the percentage change. Week "1" represents the acclimatization period, while Weeks 2 to 4 correspond to the active administration of *C. minuta*, followed by the observation period from Weeks 5 to 12. Comparisons of body weight gain were conducted between groups using data from Week 4 to assess the effects of *C. minuta*, as the final administration occurred in Week 4, rendering the body weight changes in Week 5 particularly significant. Additionally, long-term effects were evaluated between groups using data from Week 12.

Body Weight Gain Data

Female groups bodyweight (g)

Period	Acclimatisation		C. minuta administration period						Follow-up period															
Weeks	1		2		3		4		5		6		7		8		9		10		11		12	
Female groups	Mean	±SD	Mean	±SD	Mean	±SD	Mean	±SD	Mean	±SD	Mean	±SD	Mean	±SD	Mean	±SD	Mean	±SD	Mean	±SD	Mean	±SD	Mean	±SD
ND	24.20	34.10	25.50	36.00	26.10	36.20	28.10	37.90	29.20	38.10	29.20	38.60	30.10	39.50	31.00	40.60	31.70	40.80	30.40	41.60	32.50	41.60	32.40	41.80
ND + CM	27.00	36.30	26.50	36.20	29.60	38.80	30.10	40.00	31.40	41.20	31.80	42.30	32.80	42.40	32.70	42.80	33.50	43.50	34.60	44.50	31.60	42.30	35.50	45.00
ND + AB + CM	25.50	35.90	26.50	37.20	27.60	38.10	28.70	39.60	27.30	38.50	29.40	41.70	30.90	41.70	31.40	43.40	32.20	44.60	31.90	45.60	33.30	46.50	33.00	46.00
HFD	26.10	35.10	27.60	37.20	28.80	38.10	29.70	38.50	31.20	39.50	31.70	40.00	32.10	40.30	33.90	41.80	34.40	41.78	36.00	41.67	35.40	41.89	34.60	40.38
HFD + CM	25.00	33.10	24.50	32.50	27.10	33.70	27.80	35.50	28.50	36.50	29.40	36.90	30.20	38.40	30.30	38.90	31.30	39.20	31.80	40.30	30.90	36.40	31.10	40.10
HFD + AB + CM	26.50	35.20	27.40	36.80	28.40	37.60	29.80	38.70	29.80	36.50	31.40	40.50	30.70	41.70	32.00	43.20	32.90	42.60	34.00	43.90	34.40	45.10	36.00	45.20
SD	25.50	32.90	27.30	34.40	28.40	36.10	30.30	37.30	30.70	38.00	31.60	38.60	32.50	39.60	34.00	40.20	34.80	40.20	35.70	41.00	36.20	42.20	36.60	41.90
SD + CM	24.20	33.80	24.80	32.90	26.20	36.70	27.20	38.00	28.20	38.90	28.80	39.60	29.40	40.80	28.60	42.00	30.00	42.10	29.90	42.80	28.50	40.40	26.00	43.90
SD + AB + CM	25.70	32.80	27.00	34.00	28.70	35.40	30.00	36.30	29.00	35.20	30.44	37.30	31.33	38.90	32.22	40.00	31.89	40.10	33.00	40.20	32.11	41.20	33.11	42.80

Female groups bodyweight gain (%)

Period	Acclimatisation		C. minuta administration period						Follow-up period															
Weeks	1		2		3		4		5		6		7		8		9		10		11		12	
Female groups	Mean	±SD	Mean	±SD	Mean	±SD	Mean	±SD	Mean	±SD	Mean	±SD	Mean	±SD	Mean	±SD	Mean	±SD	Mean	±SD	Mean	±SD	Mean	±SD
ND	0.00		5.40	3.80	8.00	7.06	15.20	5.59	20.50	5.89	20.60	4.29	24.30	6.16	28.10	7.59	30.90	8.14	25.50	5.43	34.40	9.74	33.90	8.86
ND + CM	0.00		-1.60	4.69	9.80	4.64	11.70	4.28	16.50	5.71	18.00	8.30	21.70	6.74	21.30	7.51	24.50	9.46	28.50	10.87	17.20	7.74	31.50	12.05
ND + AB + CM	0.00		3.90	3.15	8.20	4.40	12.00	3.89	7.00	4.94	15.60	12.60	21.10	5.36	23.10	7.01	26.20	9.29	25.10	6.06	30.50	6.63	29.30	11.06
HFD	0.00		5.90	3.57	10.60	5.76	13.10	6.45	19.70	5.99	21.70	7.59	23.40	9.15	30.30	15.49	32.10	8.34	38.40	14.51	36.00	18.28	32.50	14.39
HFD + CM	0.00		-1.90	4.22	8.40	5.10	11.40	2.29	14.10	4.60	17.60	6.02	20.60	10.45	21.30	7.84	25.30	6.64	27.20	8.12	23.70	9.26	24.50	8.00
HFD + AB + CM	0.00		3.40	3.19	7.20	7.12	11.90	5.30	12.60	13.29	18.60	5.21	15.80	6.16	20.40	8.56	23.90	6.94	27.90	11.39	29.40	10.97	35.40	13.69
SD	0.00		6.50	4.36	10.07	5.15	15.69	5.09	16.77	5.30	19.12	5.08	21.18	5.40	24.66	5.84	26.49	5.25	28.11	6.09	29.18	5.73	29.71	7.19
SD + CM	0.00		2.01	8.21	7.22	7.23	10.79	6.34	14.00	5.32	15.57	7.26	17.43	5.61	14.75	8.40	18.86	7.23	18.55	8.14	14.72	6.81	21.15	7.08
SD + AB + CM	0.00		4.38	5.54	10.29	3.60	11.95	5.58	10.39	6.05	14.62	6.17	17.21	4.57	19.43	4.55	18.15	8.41	21.09	5.99	18.80	8.45	21.32	7.81

Male groups bodyweight (g)

Period	Acclimatisation		C. minuta administration period						Follow-up period															
Weeks	1		2		3		4		5		6		7		8		9		10		11		12	
Female groups	Mean	±SD	Mean	±SD	Mean	±SD	Mean	±SD	Mean	±SD	Mean	±SD	Mean	±SD	Mean	±SD	Mean	±SD	Mean	±SD	Mean	±SD	Mean	±SD
ND	34.10	2.64	36.00	1.94	36.20	2.90	37.90	2.38	38.10	1.91	38.60	2.01	39.50	2.27	40.60	2.27	40.80	2.44	41.60	2.76	41.60	2.88	41.80	2.90
ND + CM	36.30	1.06	36.20	1.75	38.80	1.55	40.00	1.25	41.20	1.62	42.30	1.49	42.40	1.90	42.80	1.40	43.50	1.84	44.50	1.78	42.30	1.95	45.00	1.89
ND + AB + CM	35.90	3.70	37.20	3.26	38.10	2.85	39.60	3.41	38.50	3.44	41.70	2.75	41.70	3.06	43.40	2.95	44.60	2.80	45.60	2.80	46.50	3.50	46.00	3.71
HFD	35.10	2.85	37.20	3.36	38.10	2.64	38.50	3.10	39.50	2.46	40.00	2.05	40.30	2.31	41.80	2.49	41.78	3.03	41.67	3.20	41.89	3.79	40.38	2.33
HFD + CM	33.10	2.92	32.50	3.95	33.70	3.74	35.50	3.60	36.50	3.41	36.90	3.31	38.40	3.89	38.90	4.36	39.20	5.18	40.30	4.69	36.40	4.97	40.10	4.82
HFD + AB + CM	35.20	1.99	36.80	1.87	37.60	2.27	38.70	2.31	36.50	2.46	40.50	2.68	41.70	2.75	43.20	2.78	42.60	2.67	43.90	3.90	45.10	3.81	45.20	3.58
SD	32.90	2.28	34.40	2.84	36.10	2.77	37.30	3.02	38.00	3.46	38.60	2.46	39.60	2.27	40.20	3.01	40.20	2.04	41.00	3.86	42.20	3.68	41.90	4.18
SD + CM	33.80	1.23	32.90	1.66	36.70	1.89	38.00	2.26	38.90	2.33	39.60	2.12	40.80	2.49	42.00	2.40	42.10	3.21	42.80	2.82	40.40	2.12	43.90	2.42
SD + AB + CM	32.80	2.15	34.00	2.16	35.40	2.72	36.30	2.91	35.20	2.97	37.30	2.31	38.90	2.33	40.00	2.79	40.10	2.28	40.20	2.78	41.20	3.08	42.80	3.65

Male groups bodyweight gain (%)

Period	Acclimatisation		C. minuta administration period						Follow-up period															
Weeks	1		2		3		4		5		6		7		8		9		10		11		12	
Female groups	Mean	±SD	Mean	±SD	Mean	±SD	Mean	±SD	Mean	±SD	Mean	±SD	Mean	±SD	Mean	±SD	Mean	±SD	Mean	±SD	Mean	±SD	Mean	±SD
ND	0.00		5.80	3.10	6.20	4.93	10.60	3.62	12.00	5.07	13.50	6.01	16.20	7.56	19.40	6.30	20.00	8.50	22.20	6.05	22.20	7.48	22.90	8.08
ND + CM	0.00		-0.20	5.15	6.90	2.97	10.20	2.60	13.50	4.56	16.50	2.60	16.80	3.42	17.90	3.10	19.80	4.11	22.60	3.60	16.50	4.10	24.00	4.44
ND + AB + CM	0.00		3.80	2.90	6.50	5.68	10.10	4.37	7.40	3.67	16.60	5.96	16.70	8.19	21.40	7.93	24.90	9.81	27.80	10.62	30.10	10.31	28.70	9.46
HFD	0.00		6.00	3.58	8.70	3.82	9.10	1.77	12.70	4.19	14.20	4.86	15.00	4.48	19.30	4.22	19.90	3.51	19.60	5.18	20.10	6.60	18.80	6.33
HFD + CM	0.00		-1.90	6.88	1.90	7.94	7.30	4.12	10.30	4.35	11.60	4.91	16.00	4.45	17.40	4.60	18.10	6.88	21.50	4.99	9.60	6.96	21.00	7.58
HFD + AB + CM	0.00		4.60	2.60	6.90	4.33	9.50	4.03	3.80	5.42	15.10	5.84	18.50	5.80	22.80	6.81	21.20	8.04	24.70	9.01	28.20	9.85	24.00	10.84
SD	0.00		4.11	6.05	8.66	5.68	11.58	5.17	13.16	4.90	14.67	4.88	16.82	5.20	18.00	4.84	18.10	4.86	19.43	5.66	21.75	5.75	21.01	7.04
SD + CM	0.00		-2.84	3.28	7.80	2.90	10.91	3.31	12.98	2.91	14.57	1.93	17.02	3.04	19.41	2.70	19.48	3.68	20.86	3.28	16.26	1.95	22.92	1.98
SD + AB + CM	0.00		3.49	2.97	7.22	3.25	9.52	2.83	7.01	3.78	11.80	3.76	15.58	3.72	17.87	3.28	17.94	3.05	18.09	3.49	20.04	3.26	23.03	4.26

Figure S4. Mean body weight (g) and body weight gain (%) of female and male groups over the entire study period.

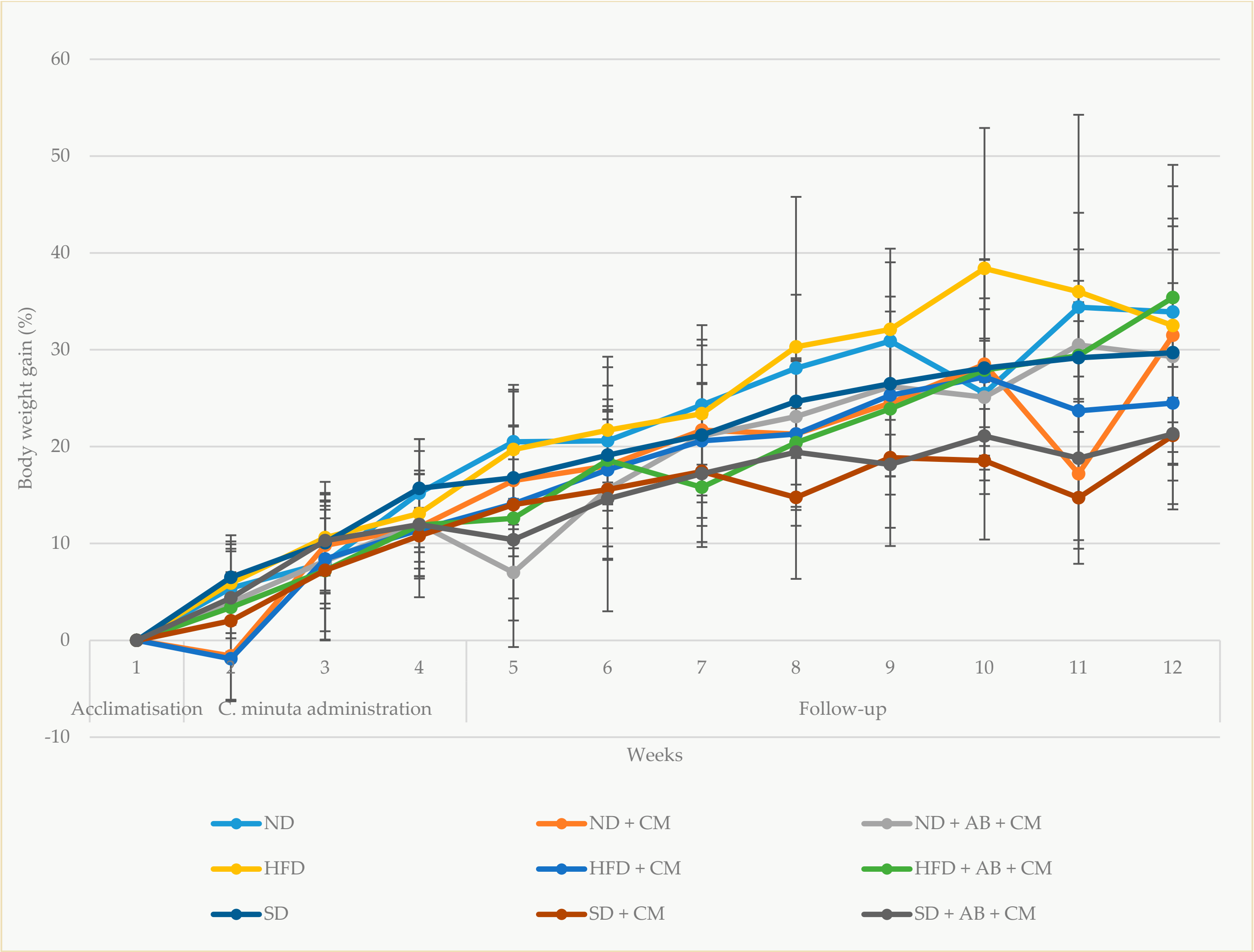

Figure S5. Mean body weight (g) and body weight gain (%) of female groups over the entire study period (n = 89).

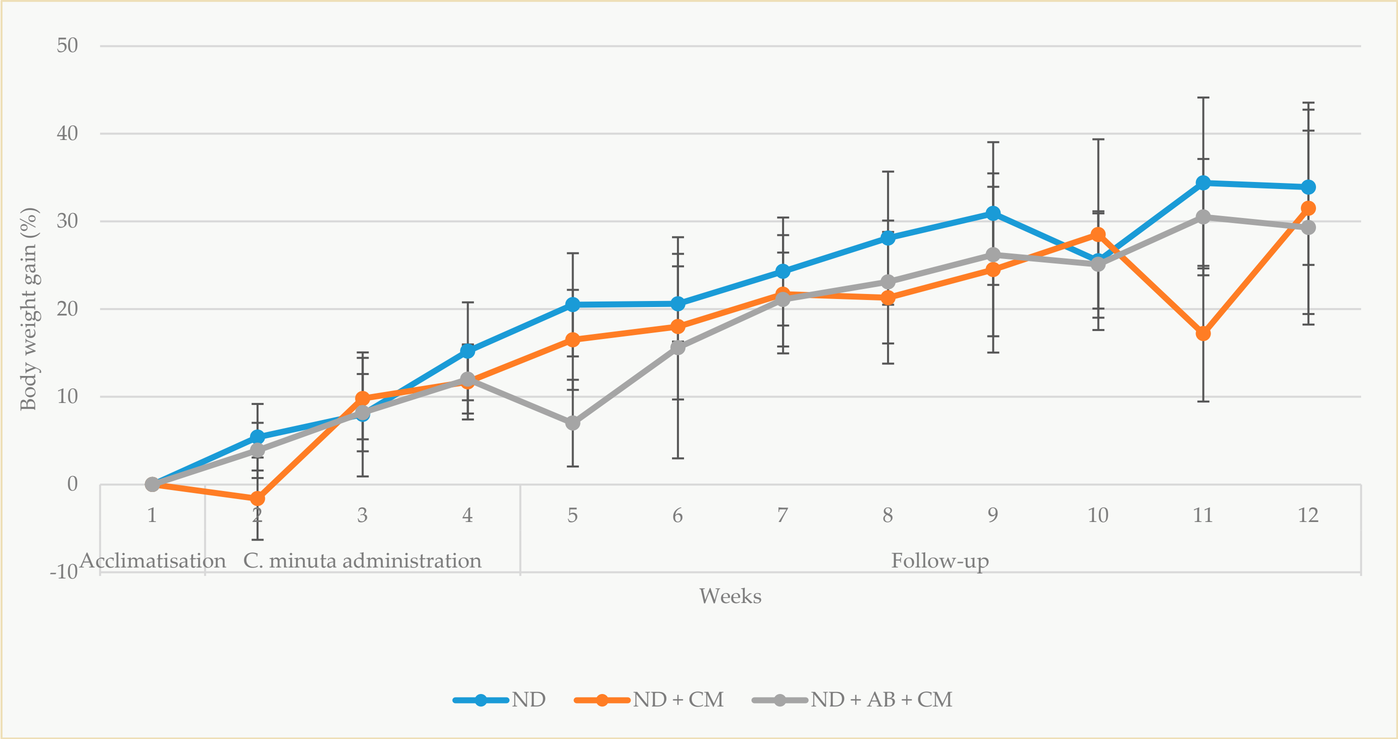

Figure S6. Body weight changes (%) in male groups fed a normal diet (ND) (n = 30).

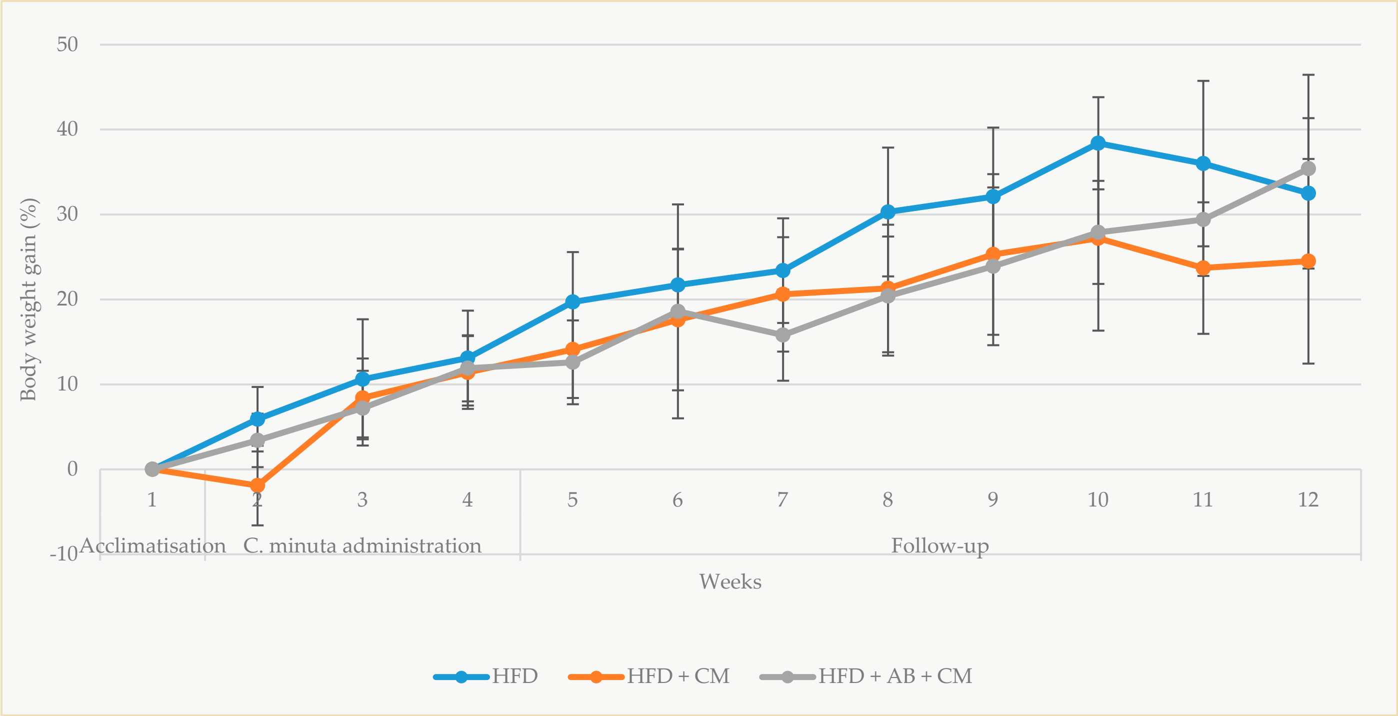

Figure S7. Body weight changes (%) in female HFD groups (n = 30).

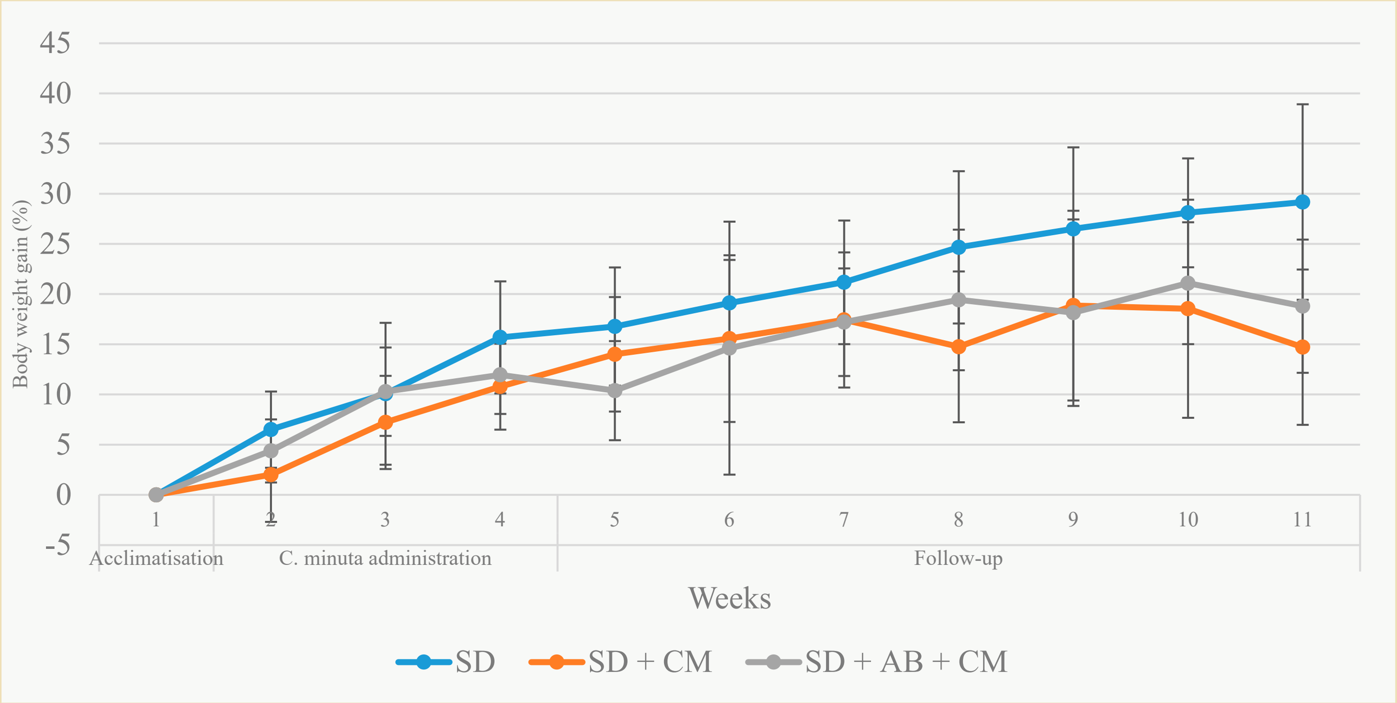

Figure S8. Body weight changes (%) in female mice consuming sweetener-containing fluid (SD) (n = 29).

Male body weight gain (%)

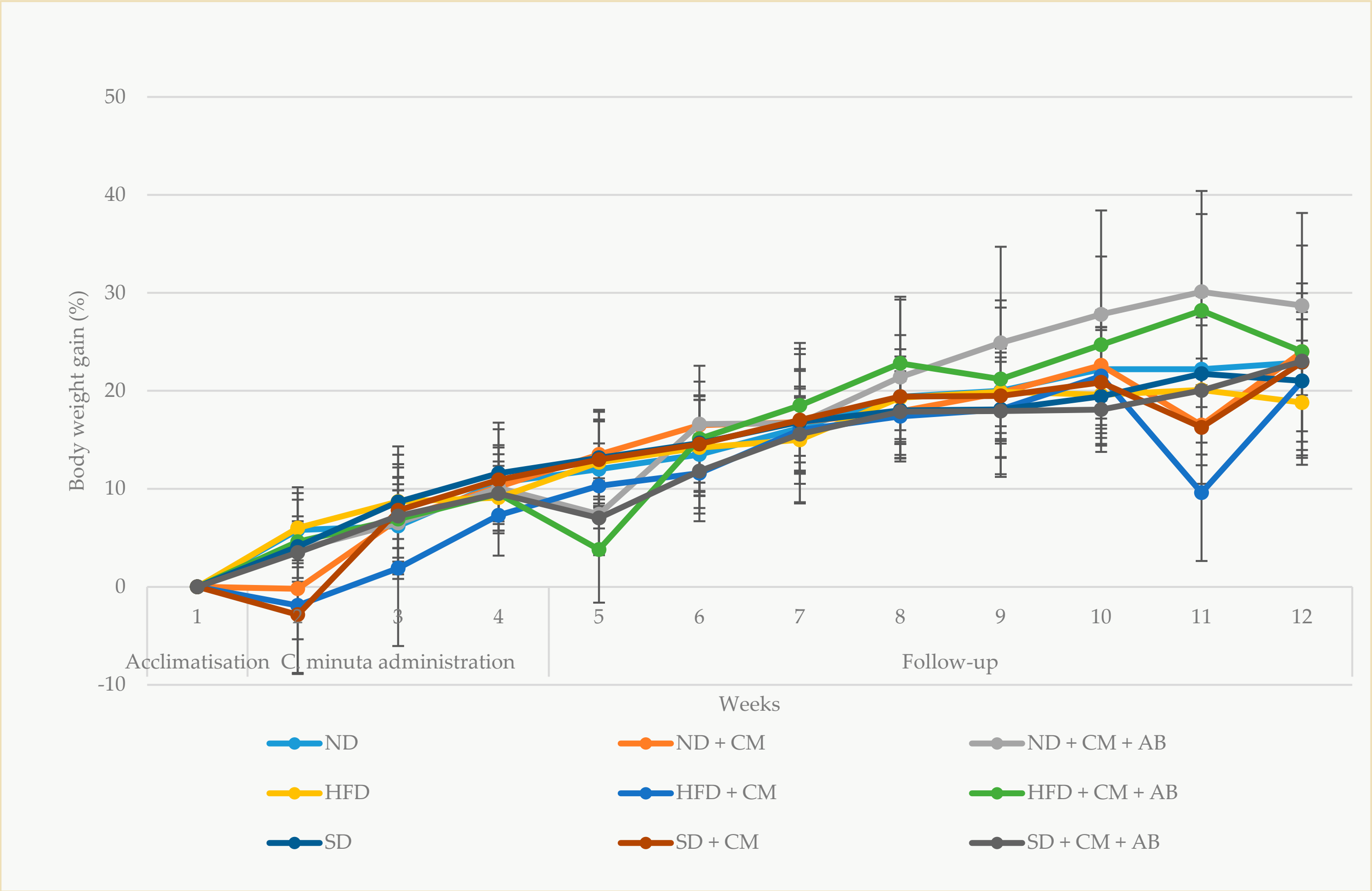

Figure S9. Mean body weight (g) and body weight gain (%) of male groups over the entire study period (n = 88).

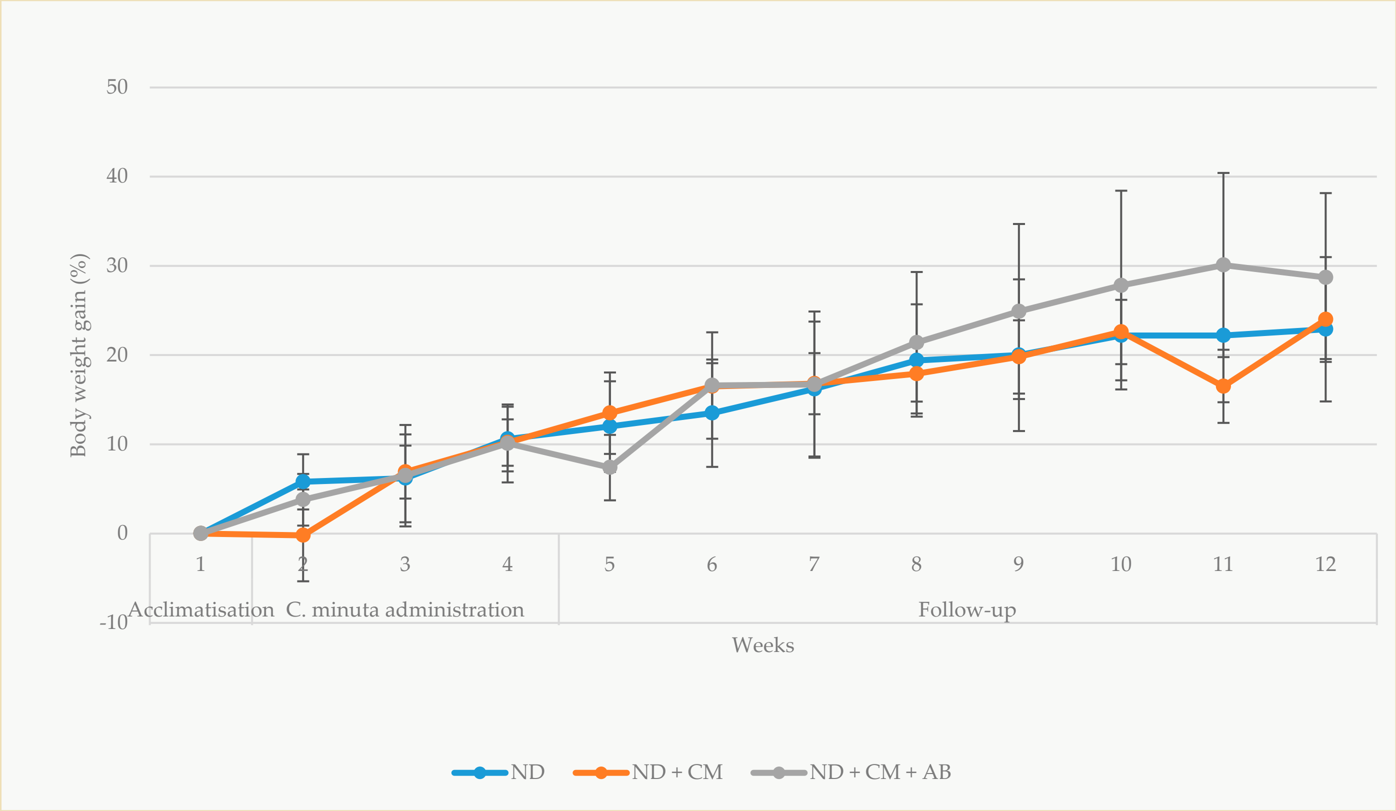

Figure S10. Body weight changes (%) in male mice fed a normal diet (ND) (n = 30).

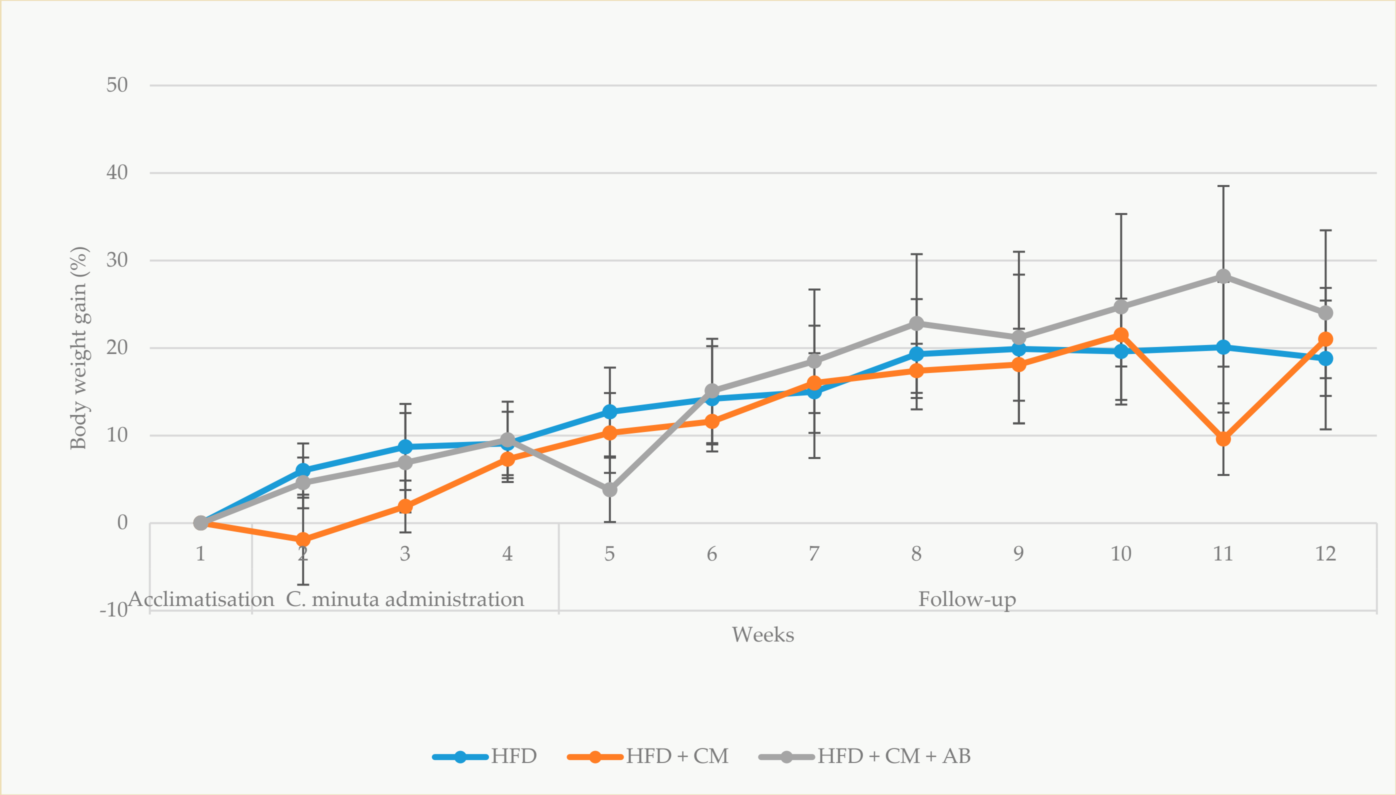

Figure S11. Body weight changes (%) in male mice fed a high-fat diet (HFD) (n = 28).

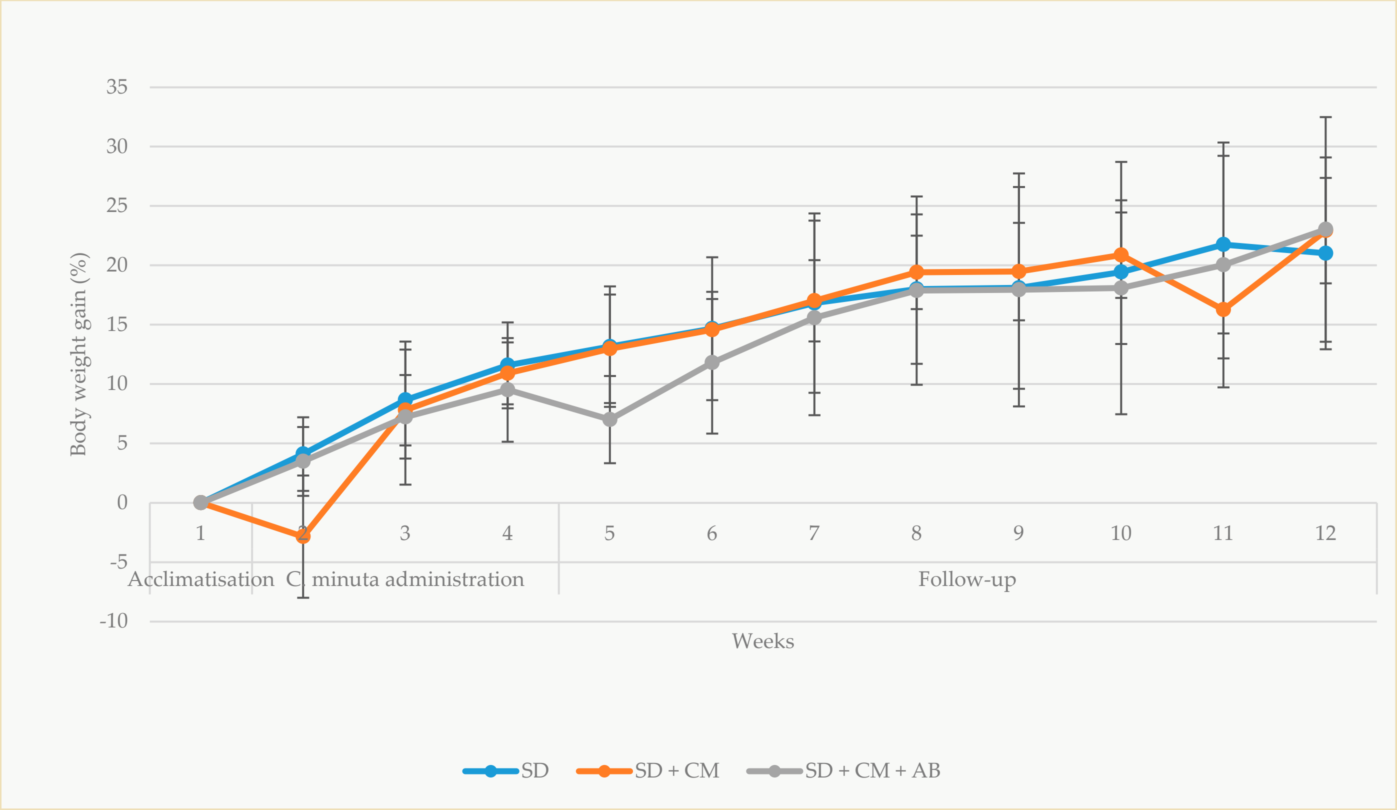

Figure S12. Body weight changes (%) in male mice consuming sweetener-containing fluid (SD) (n = 30).

Energy intake measurement

Female groups energy intake (kcal)																					
Period	Acclimatisation	C. minuta administration						Follow-up													
Weeks	1	2		3		4		5		6		7		8		9		10		11	
Female groups		Mean	±SD	Mean	±SD	Mean	±SD	Mean	±SD	Mean	±SD	Mean	±SD	Mean	±SD	Mean	±SD	Mean	±SD	Mean	±SD
**ND**	0	212.85	36.49	557.93	9.12	454.29	265.14	625.65	36.49	635.33	18.24	582.11	11.40	719.18	141.39	472.46	6.84	533.74	2.28	546.64	25.08
**ND + CM**	0	295.09	11.40	674.03	9.12	665.96	47.89	748.20	31.93	743.36	11.40	725.63	0.00	765.94	66.13	615.98	45.61	722.40	54.73	370.88	54.73
**ND + AB + CM**	0	278.96	6.84	528.90	9.12	604.69	11.40	580.50	9.12	611.14	25.08	572.44	20.52	604.69	38.77	469.24	11.40	533.74	11.40	470.85	54.73
**HFD**	0	235.60	8.77	626.20	39.46	565.75	24.11	621.55	54.80	649.45	19.73	547.15	59.18	671.15	10.96	429.35	59.18	581.25	15.34	564.20	113.99
**HFD + CM**	0	215.45	15.34	435.55	50.42	514.60	35.07	531.65	37.26	534.75	63.57	500.65	28.50	578.15	50.42	444.85	32.88	513.05	6.58	333.25	2.19
**HFD + AB + CM**	0	296.05	37.26	522.35	46.03	561.10	17.54	613.80	74.53	564.20	52.61	544.05	10.96	602.95	24.11	463.45	41.65	582.80	0.00	413.85	41.65
**SD**	0	249.94	11.40	601.46	20.52	646.61	6.84	683.70	54.73	693.38	18.24	632.10	18.24	719.18	22.80	503.75	10.96	628.88	13.68	641.78	4.56
**SD + CM**	0	387.00	159.63	1106.18	488.01	1051.35	442.40	986.85	232.60	904.61	212.08	799.80	22.80	835.28	31.93	601.40	96.45	759.49	143.67	435.38	18.24
**SD + AB + CM**	0	328.95	22.80	712.73	50.17	632.10	82.10	659.51	93.50	670.80	127.70	672.41	129.98	757.88	182.43	537.85	89.87	680.48	123.14	548.25	31.93

Male groups energy intake (kcal)																					
Period	Acclimatisation	C. minuta administration						Follow-up													
Weeks	1	2		3		4		5		6		7		8		9		10		11	
Male groups		Mean	±SD	Mean	±SD	Mean	±SD	Mean	±SD	Mean	±SD	Mean	±SD	Mean	±SD	Mean	±SD	Mean	±SD	Mean	±SD
**ND**	0	285.41	4.95	706.28	5.66	741.75	1.41	786.90	9.90	753.04	2.12	719.18	4.24	820.76	0.71	604.69	3.54	704.66	4.95	709.50	7.07
**ND + CM**	0	309.60	1.41	674.03	8.49	746.59	7.78	777.23	0.00	788.51	10.61	775.61	16.26	883.65	7.07	682.09	6.36	785.29	17.68	435.38	7.07
**ND + AB + CM**	0	311.21	7.78	682.09	28.99	715.95	26.87	706.28	22.63	728.85	14.14	699.83	21.21	815.93	36.77	582.11	16.26	712.73	29.70	561.15	31.11
**HFD**	0	296.05	2.12	651.00	5.66	672.70	21.21	703.70	22.63	691.30	33.94	678.90	39.60	796.70	33.94	554.90	1.41	658.75	16.26	695.95	7.78
**HFD + CM**	0	285.20	4.24	593.65	2.12	620.00	1.41	652.55	6.36	620.00	14.14	620.00	18.38	713.00	16.97	522.35	4.95	601.40	8.49	353.40	2.83
**HFD + AB + CM**	0	313.10	9.90	587.45	13.44	647.90	19.80	647.90	5.66	624.65	2.12	630.85	3.54	719.20	7.07	483.55	26.16	649.45	4.95	520.80	2.83
**SD**	0	317.66	2.12	765.94	6.36	865.91	6.36	875.59	14.85	862.69	20.51	830.44	14.85	944.93	8.49	697.50	7.07	635.33	84.85	925.58	59.40
**SD + CM**	0	314.44	9.19	685.31	21.92	699.83	8.49	778.84	10.61	764.33	2.83	724.01	3.54	801.41	6.36	592.10	7.07	754.65	8.49	553.09	31.82
**SD + AB + CM**	0	366.04	21.92	812.70	52.33	827.21	44.55	790.13	29.70	769.16	7.78	746.59	10.61	891.71	17.68	640.15	6.36	765.94	7.78	595.01	6.36

Figure S13. Mean energy intake (kcal) of female and male groups over the entire study period.

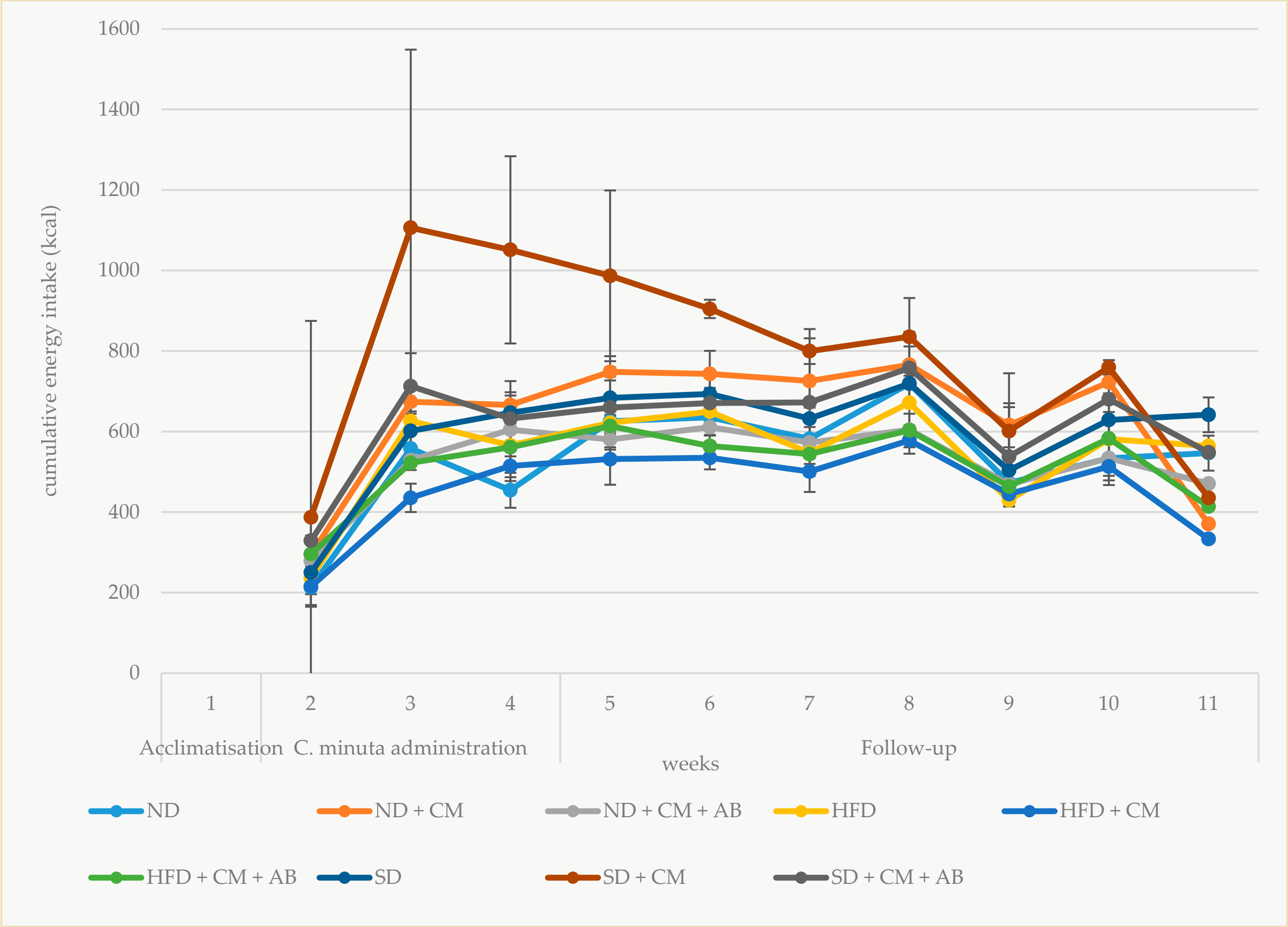

Figure S14. Cumulative mean energy intake (kcal) of female groups over the entire study period (n = 89).

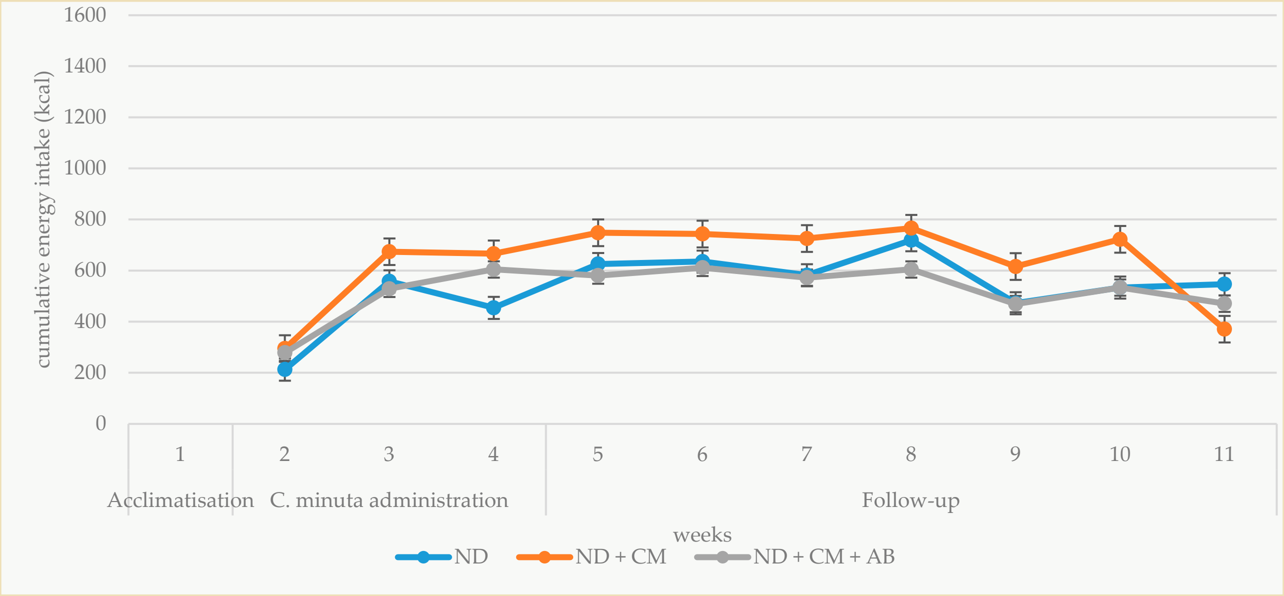

Figure S15. Cumulative energy intake (kcal) in female groups fed a normal diet (ND) (n = 30).

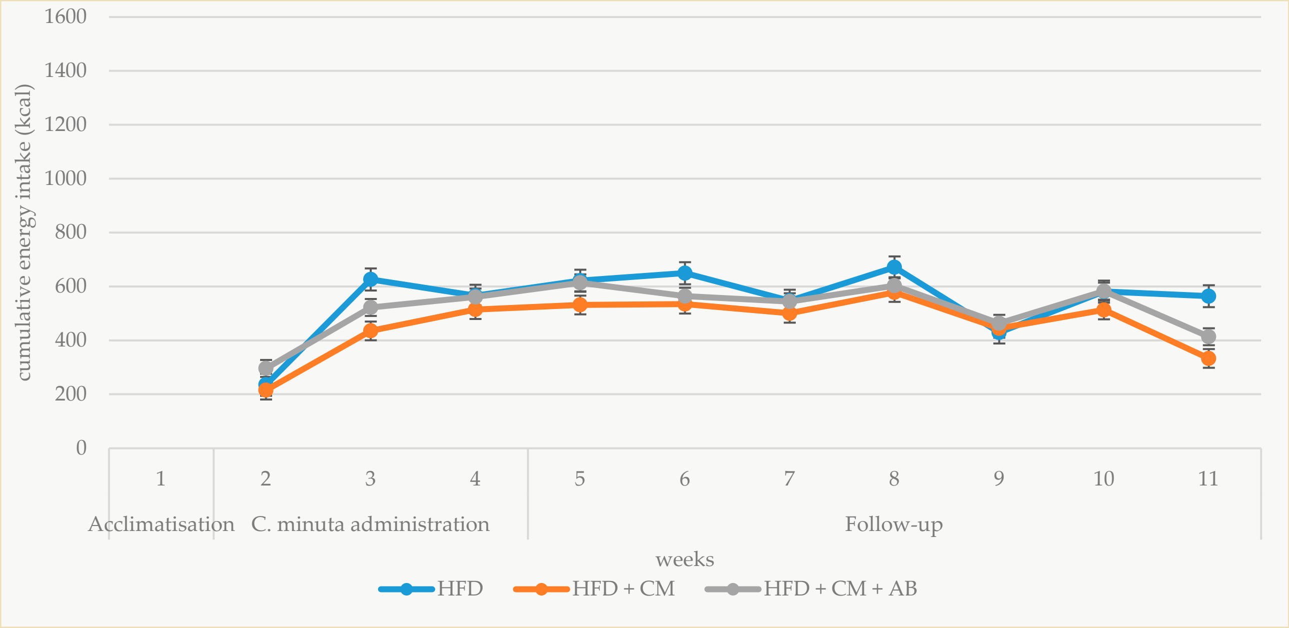

Figure S16. Cumulative energy intake (kcal) in female groups fed a high-fat diet (HFD) (n = 30).

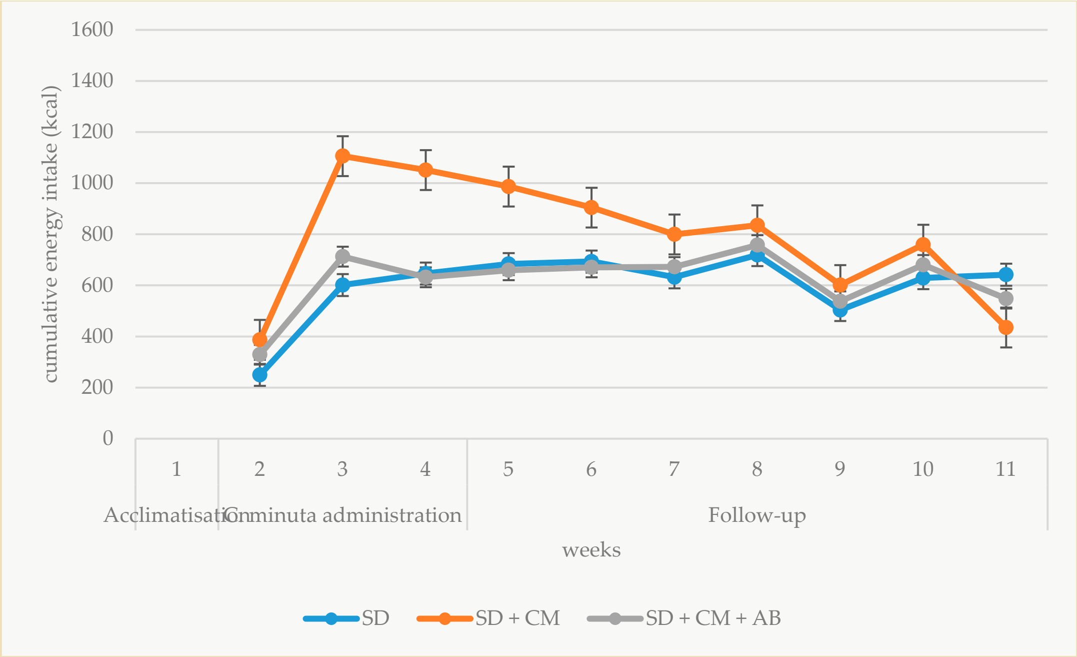

Figure S17. Cumulative energy intake (kcal) in female groups consuming sweetener-containing fluid (SD) (n = 29).

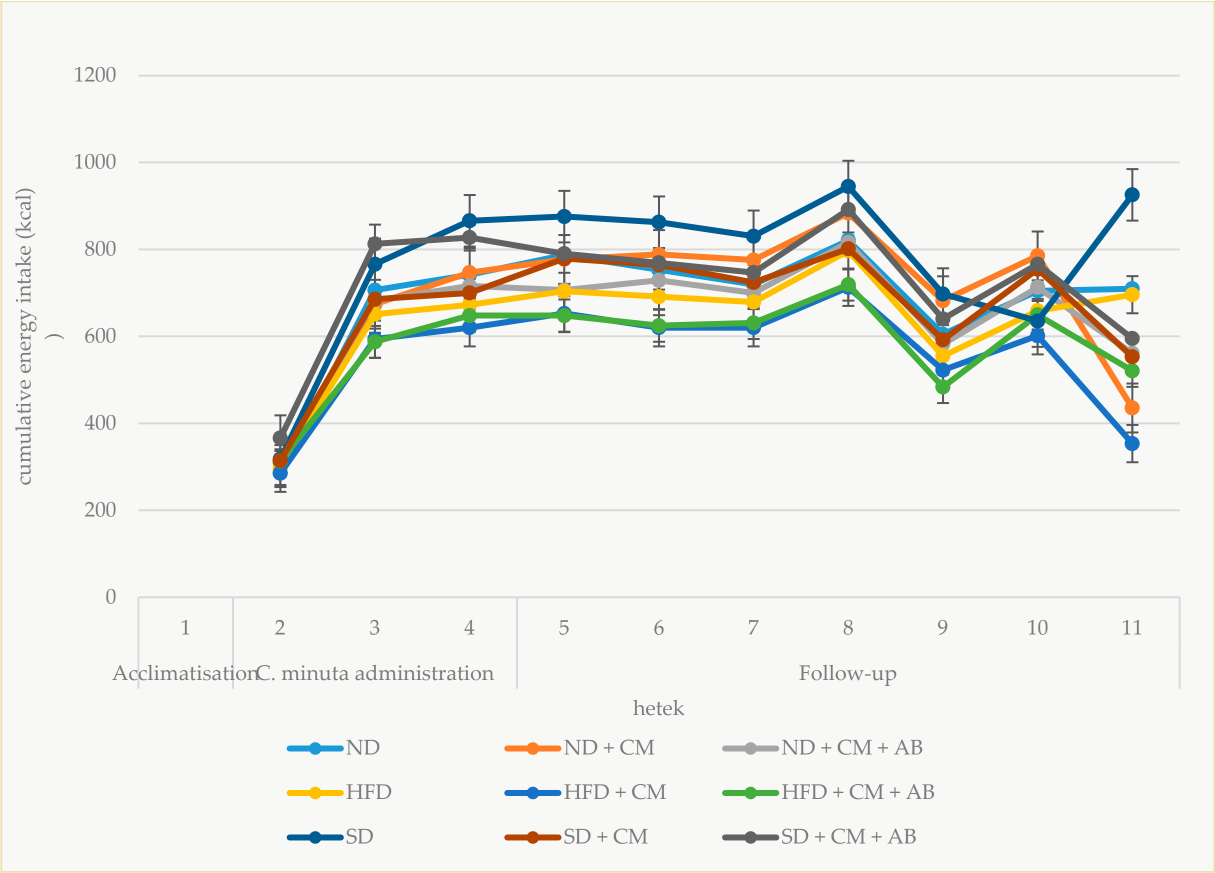

Figure S18. Cumulative mean energy intake (kcal) of male groups over the entire study period (n = 88).

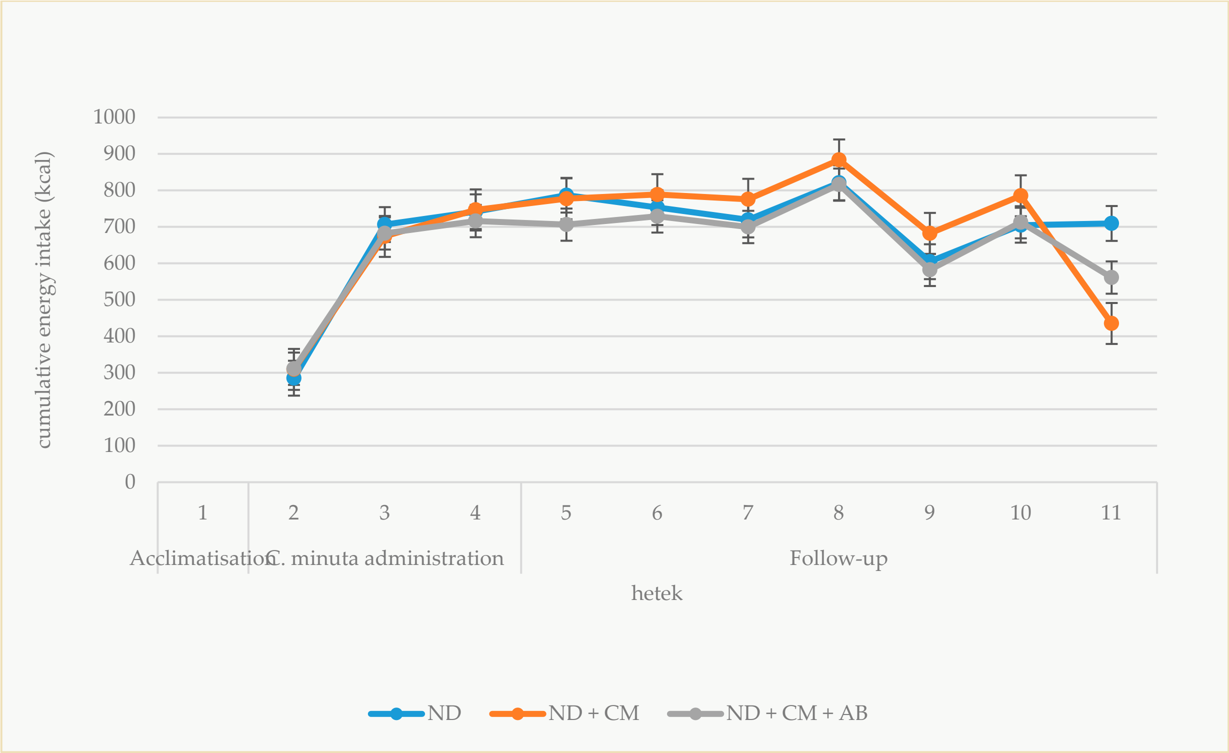

Figure S19. Cumulative energy intake (kcal) in male groups fed a normal diet (ND) (n = 30).

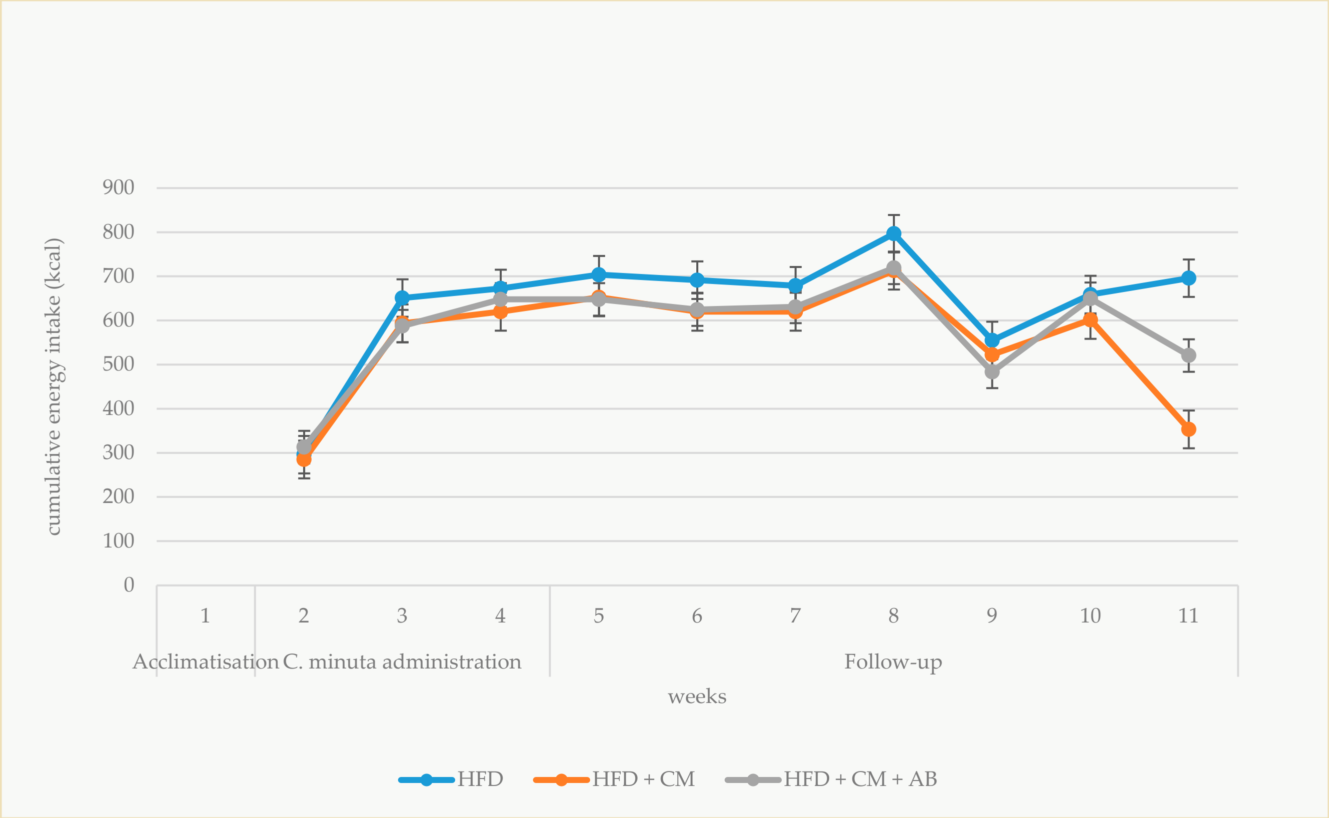

Figure S20. Cumulative energy intake (kcal) in male groups fed a high-fat diet (HFD) (n = 28).

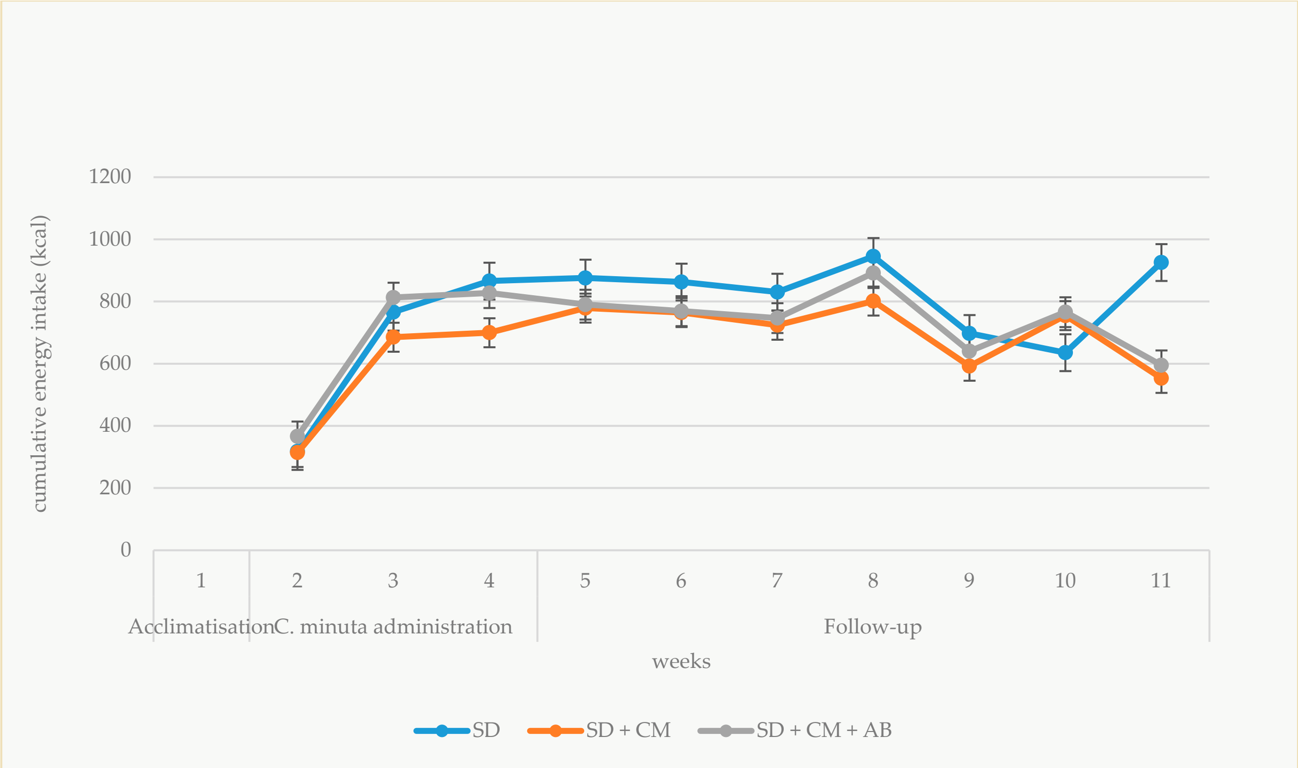

Figure S21. Cumulative energy intake (kcal) in male groups consuming sweetener-containing fluid (SD) (n = 30)
